# Ir(III) Metal Emitters with Cyano‐Modified Imidazo[4,5‐b]pyridin‐2‐ylidene Chelates for Deep‐Blue Organic Light‐Emitting Diodes

**DOI:** 10.1002/advs.202309389

**Published:** 2024-04-30

**Authors:** Yixin Wu, Yangyang Xin, Yi Pan, Shek‐Man Yiu, Jie Yan, Kai Chung Lau, Lian Duan, Yun Chi

**Affiliations:** ^1^ Department of Chemistry Department of Materials Science and Engineering Center of Super‐Diamond and Advanced Films (COSDAF) City University of Hong Kong Hong Kong SAR 999077 China; ^2^ Key Laboratory of Organic Optoelectronics and Molecular Engineering of Ministry of Education Department of Chemistry Tsinghua University Beijing 100084 China

**Keywords:** blue, carbene, cyclometalates, iridium, organic light‐emitting diodes

## Abstract

Ir(III) carbene complexes have been explored as one of the best blue phosphors for their high performance. Herein, the authors designed and synthesized a series of blue‐emitting Ir(III) phosphors (*f*‐ct9a–c), featuring *fac*‐coordinated cyano‐imidazo[4,5‐b]pyridin‐2‐ylidene cyclometalates. These Ir(III) complexes exhibit true‐blue emission with a peak maximum spanning 448–467 nm, with high photoluminescence quantum yields of 81–88% recorded in degassed toluene. Moreover, OLED devices bearing phosphors *f*‐ct9a and *f*‐ct9b deliver maximum external quantum efficiencies (EQE_max_) of 25.9% and 30.3%, together with Commission Internationale de L'Eclairage (CIE*_x,y_*) coordinates of (0.157, 0.225) and (0.142, 0.169), respectively. Remarkably, the *f*‐ct9b‐based device displays an incredible EQE of 29.0% at 5000 cd·m^−2^. The hyper‐OLED device based on *f*‐ct9b and ν‐DABNA exhibits an EQE_max_ of 34.7% and CIE_x,y_ coordinates of (0.122, 0.131), affirming high potentials in achieving efficient blue electroluminescence.

## Graphical abstract

A new class of blue emissive Ir(III) complexes bearing facially arranged cyano‐modified imidazo[4,5‐b]pyridin‐2‐ylidene chelates are reported, showing high emission quantum yields and short radiative lifetimes. In addition to the efficient blue electrophosphorescence, one hyper‐OLED device delivers a narrowband blue emission with CIE_y_ coordinate of 0.123, EQE_max_ of 32.1%, and EQE of 21.3% at 5000 cd·m^−2^, respectively.

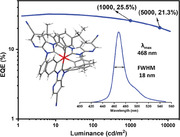

## 1 Introduction

Since the first observation of organic electroluminescence by Tang and VanSlyke in 1987,^[^ ^1^ ^]^ organic light‐emitting diodes (OLEDs) have rapidly evolved and turned into an indispensable technology of the 21^st^ century, particularly in serving as either the displays or monitors of various electronic appliances.^[^ ^2^ ^]^ Of course, the organic materials that transport electrons and charges and serve as the host to confine and harvest the excitons are found to be equally important to OLEDs. Light‐emitting materials still hold the pivotal position since they control both the inherent stability and overall efficiency. Fundamentally, emitters have been classified as i) fluorescent, ii) phosphorescent, and iii) thermally activated delayed fluorescent (TADF) emitters, respectively. It is known to the field that fluorescent emitters can only use the electrically generated singlet excitons, which limits its maximal internal quantum efficiency (IQE) to only 25%. In contrast, both phosphorescent and TADF emitters are capable of harvesting both the singlet and triplet excitons via heavy atom‐induced spin‐orbit coupling or fast reverse intersystem crossing governed by substantially reduced *ΔE_ST_* in achieving a full theoretical IQE of 100%, making the last two emitters an ideal candidate for commercial OLED applications.^[^ ^3^ ^]^

Next, regarding the color hue, blue emission possesses the shortest emission wavelength and highest energy in comparison to other elementary colors, that is, red and green. This makes the blue emitters less stable than the red and green ones, due to the higher internal energy of the molecules incurred at their excited states. Moreover, this high energy also fosters a faster emission quenching, via vibration‐induced internal conversion or thermal population to the higher‐lying quenching states, both were afforded an inferior quantum yield for blue emission. These properties make the design and preparation of blue emitters a very challenging and important task.

**Scheme 1:**
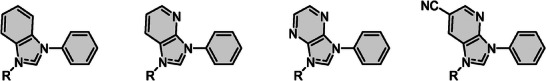
Representative structures of benzo[d]imidazol‐2‐ylidene, imidazo[4,5‐b]pyridin‐2‐ylidene, imidazo[4,5‐b]pyrazin‐2‐ylidene, and 6‐cyano‐imidazo[4,5‐b]pyridin‐2‐ylidene cyclometalates, from left to right.

Among all phosphorescent emitters, Ir(III) complexes with functional pyridinyl and quinolinyl cyclometalates were already used for their green and red luminescence in commercial applications. However, judging from the basis of coordination chemistry, homoleptic Ir(III) complexes bearing solely the carbene cyclometalates should be the best choice for blue phosphorescence due to the presence of six strongest Ir–C bonding interactions.^[^ ^4^ ^]^ Hence, structure‐property relationships were thoroughly examined for relevant Ir(III) complexes bearing benzo[d]imidazol‐2‐ylidene,^[^ ^5^ ^]^ imidazo[4,5‐b]pyridin‐2‐ylidene,^[^ ^6^ ^]^ and imidazo[4,5‐b]pyrazin‐2‐ylidene cyclometalates (**Scheme** **1**).^[^ ^7^ ^]^ The lessons learned are listed below: i) Both *fac*‐ and *mer*‐ substituted Ir(III) complexes with parent benzo[d]imidazol‐2‐ylidene chelates, that is, those without additional electron deficient group on carbene, showed high radiation energy gap in purple region that induced by the highly destabilized LUMO energy level. ii) Only the *m*‐ Ir(III) complexes with imidazo[4,5‐b]pyridin‐2‐ylidene, that is, those with one electronegative N atom, were suitable for the fabrication of blue OLEDs, as the corresponding *fac*‐isomers tend to exhibit purple emission with higher energy.^[^ ^6a^ ^]^ iii) Imidazo[4,5‐b]pyrazin‐2‐ylidene based Ir(III) phosphors, that is, *fac*‐arranged Ir(cb)_3_ in particular, possess two skeletal N atoms, giving optimal photophysical characteristics as demanded by the benchmarked blue phosphors.^[^ ^7a^ ^]^

With these precedents, we decided to engage in the design and synthesis of a cyano‐modified imidazo[4,5‐b]pyridin‐2‐ylidene chelate and corresponding Ir(III) derivatives. It is expected that the cyano (or carbonitrile) group possesses electron‐withdrawing properties comparable to the N skeletal atom of heterocyclic aromatics but without its enhanced basicity. Hence, these Ir(III) emitters should possess photophysical and electro‐optical properties comparable or even superior to those exhibited by Ir(cb)_3_ or derivatives. Particularly, this work represented a breakthrough in the designs, syntheses, and isolation of blue emissive Ir(III) phosphors in comparison to the information found in the public domain,^[^ ^8^ ^]^ and our approaches are elaborated below.

## 2 Results and Discussion

### 2.1 Syntheses of Chelates and Ir(III) Complexes

**Scheme 2:**
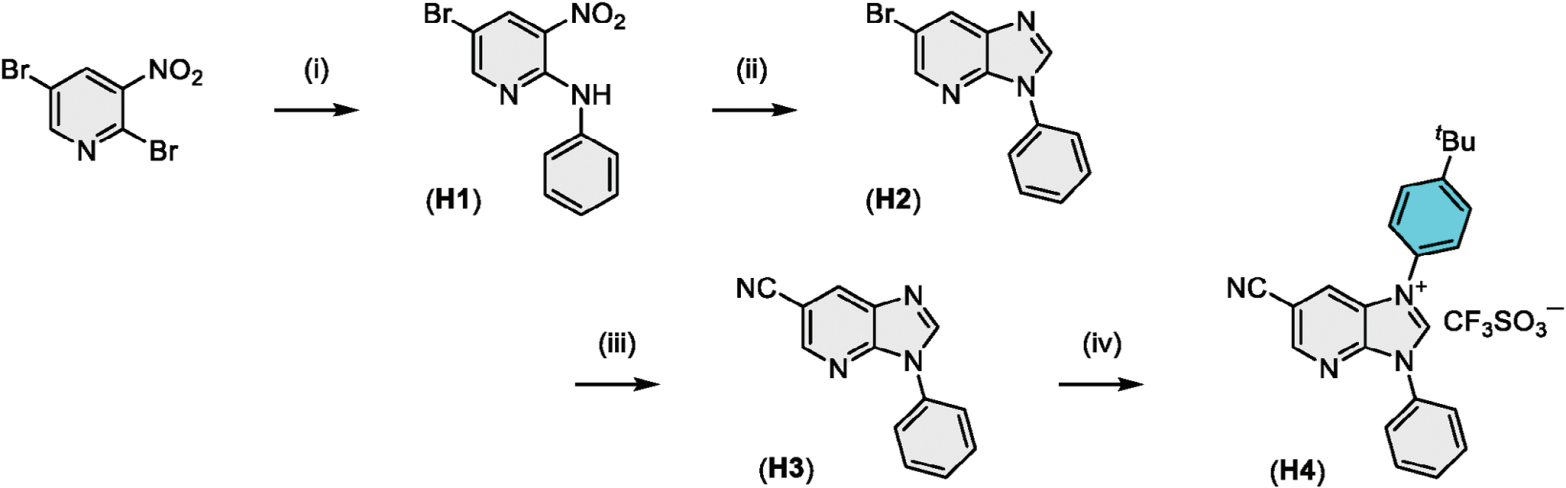
Synthesis of 6‐cyano‐3*H*‐imidazo[4,5‐*b*]pyridinium pro‐chelate; experimental conditions: i) Aniline, NEt_3_, 80 °C, ii) Fe_(s)_, HCO_2_H, 105 °C, iii) Zn(CN)_2_, Pd(PPh_3_)_4_, DMF, 120 °C, and iv) (4‐*^t^* BuC_6_H_4_I^+^Mes)(OTf^‒^), Cu_2_O, DMF, 110 °C.

There are two possible methods for the introduction of carbonitrile entity; namely: The dehydration of primary amide^[^ ^9^ ^]^ or direct halide‐to‐carbonitrile conversion.^[^ ^10^ ^]^ In the present investigation, we selected the second method to prepare the cyano functionalized imidazo[4,5‐b]pyridin‐2‐ylidene chelate, as the starting materials are much more accessible and easier to handle than that en route from amides. Consequently, as shown in **Scheme** **2**, the commercially available 2,5‐dibromo‐3‐nitropyridine was treated with aniline in the presence of triethylamine to afford 5‐bromo‐3‐nitro‐N‐phenylpyridin‐2‐amine (H1).^[^ ^11^ ^]^ Subsequent reduction of the nitro group with iron powder in formic acid gave 6‐bromo‐3‐phenyl‐3H‐imidazo[4,5‐b]pyridine (H2) directly.^[^ ^11a^ ^]^ Next, we conducted the bromide‐to‐carbonitrile substitution using zinc cyanide and Pd(PPh_3_)_4_ catalyst in giving 3‐phenyl‐3H‐imidazo[4,5‐b]pyridine‐6‐carbonitrile (H3).^[^ ^10b^ ^]^ After then, in accordance with the literature report,^[^ ^12^ ^]^ quaternization of H3 with iodonium salt (4‐*^t^* BuC_6_H_4_I^+^Mes)(OTf^‒^) and Cu_2_O catalyst yielded the demanded pro‐chelate, that is, 6‐cyano‐1‐(4‐(*tert*‐butyl)phenyl)‐3‐phenyl‐3*H*‐imidazo[4,5‐*b*]pyridin‐1‐ium (H4). Notably, all reactions exhibited satisfactory repeatability with no obvious difference between the initial and scale‐up processes. Subsequent recrystallization from diethyl ether and acetone gave a white solid, which was directly employed in making the anticipated Ir(III) carbene complexes.

**Scheme 3:**
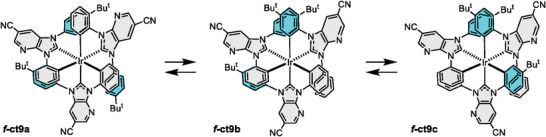
Structural drawings of isolated Ir(III) complexes *f*‐ct9a–c, to which slow equilibration occurred only in the presence of an acid catalyst and under extensive heating at high temperatures.

Next, the pro‐chelate H4 was treated with Ir(tht)_3_Cl_3_ in the presence of 10 equivalence of sodium acetate (NaOAc) as a cyclometalation promoter.^[^ ^13^ ^]^ This reaction was conducted in refluxing *o‐*dichlorobenzene (bp = 180 °C) for 24 h, affording three Ir(III) complexes *f*‐ct9a–c in an approximate ratio of 53:40:7. Their schematic drawings were depicted in **Scheme** **3**. The separation of products was typically achieved by silica gel column chromatography, followed by recrystallization from mixed solvents such as CHCl_3_ and methanol, or CH_2_Cl_2_ and methanol at RT. As expected, each of these samples undergoes catalytic isomerization in refluxing 1,2,4‐trichlorobenzene (bp = 214 °C), to which an identical distribution ratio of products versus those of their original syntheses was observed. In accordance with literature precedent,^[^ ^14^ ^]^ this transformation is best understood as the formation of a hypothetical monodentate carbene fragment bearing two non‐coordinative aryl appendages by protonation. After that, the retro‐C‐H activation is expected to afford either original or alternative cyclometalating chelate and, hence, the observed isomerization.

Notably, the higher synthetic yield of *f*‐ct9a in reference to *f*‐ct9b and *f*‐ct9c suggested that the phenyl group adjacent to the pyridinyl N atom of imidazo[4,5‐b]pyridin‐2‐ylidene fragment possesses greater reactivity. This is due to the intra‐ligand C‐H⋅⋅⋅N interaction that affords a planar local arrangement and improved cyclometalation reactivity. In contrast, the 4‐t‐butylphenyl group residing at the opposite site experienced certain van der Waals repulsion to the adjacent C–H unit, which induced a partial rotation of the 4‐t‐butylphenyl group and should be less favorable for cyclometalation. Such an intra‐chelate interaction provides a favorable control of product distribution within these Ir(III) carbene complexes.

For characterization, all Ir(III) complexes showed an identical molecular ion (M^+^) during the electrospray ionization mass spectral analysis which confirmed their isomeric nature. Notably, *f*‐ct9a possesses a ^1^H NMR spectrum with a virtual C3 symmetry, while others exhibited an asymmetric pattern involving two distinctive carbene chelates. In contrast to our previous observation,^[^ ^15^ ^]^ to which the aryl cyclometalation mainly occurred at the 4‐*t*‐butylphenyl versus phenyl appendage, complex *f*‐ct9a is assigned to possess solely the phenyl cyclometalates, while all the 4‐*t*‐butylphenyl fragments remained as the non‐coordinative pendants. This structural proposal is first confirmed by the observation of a total of four sets of doublets at δ 7.41, 6.43, 6.29, and 6.22, together with three distinctive J_HH_ coupling constants of 8.3, 2.3, and 1.9 Hz for the four aromatic protons of 4‐*t*‐butylphenyl fragments in its ^1^H NMR spectrum (ABA'B’ spin‐coupling pattern). In sharp contrast, its structural isomers, that is, *f*‐ct9b and *f*‐ct9c, displayed highly complicated spin‐spin coupling patterns that suggested the progressive replacement of phenyl with 4‐*t*‐butylphenyl cyclometalating fragments, giving an asymmetric coordination arrangement. Of course, this assignment was next confirmed by the single crystal X‐ray diffraction studies (vide infra).

Accordingly, structures of *f*‐ct9a–c were examined by single crystal X‐ray diffraction studies. As depicted in Figures S1–S3, Supporting Information, all of them display *fac*‐coordinated imidazo[4,5‐b]pyridin‐2‐ylidene cyclometalating entities. The symmetrical *f*‐ct9a exhibits three phenyl cyclometalates, while *f*‐ct9b and *f*‐ct9c possess two and one phenyl cyclometalate(s), in addition to two and one *t*‐butylphenyl appendage(s), respectively. Furthermore, the symmetrical *f*‐ct9a exhibited relatively shortened Ir–C_(carbene)_ bonds with distances (2.030(3)–2.046(3) Å) and slightly lengthened Ir–C_(aryl)_ distances (2.095(4)–2.102(4) Å), which are consistent with the common features of Ir(III) carbene complexes reported in the literature.^[^ ^7^, ^16^ ^]^ Since *f*‐ct9a displayed three identical chelates in solution at RT, the differences between individual Ir–C_(carbene)_ and C_(aryl)_ bonds, that is, 0.016(3) and 0.017(4) Å, are most likely due to the crystal packing effect occurred within the unit cells. Similarly, the metric parameters of *f*‐ct9b and *f*‐ct9c were comparable to those of *f*‐ct9a and were unremarkable.

### 2.2 Photophysical Characteristics

**Figure 1:**
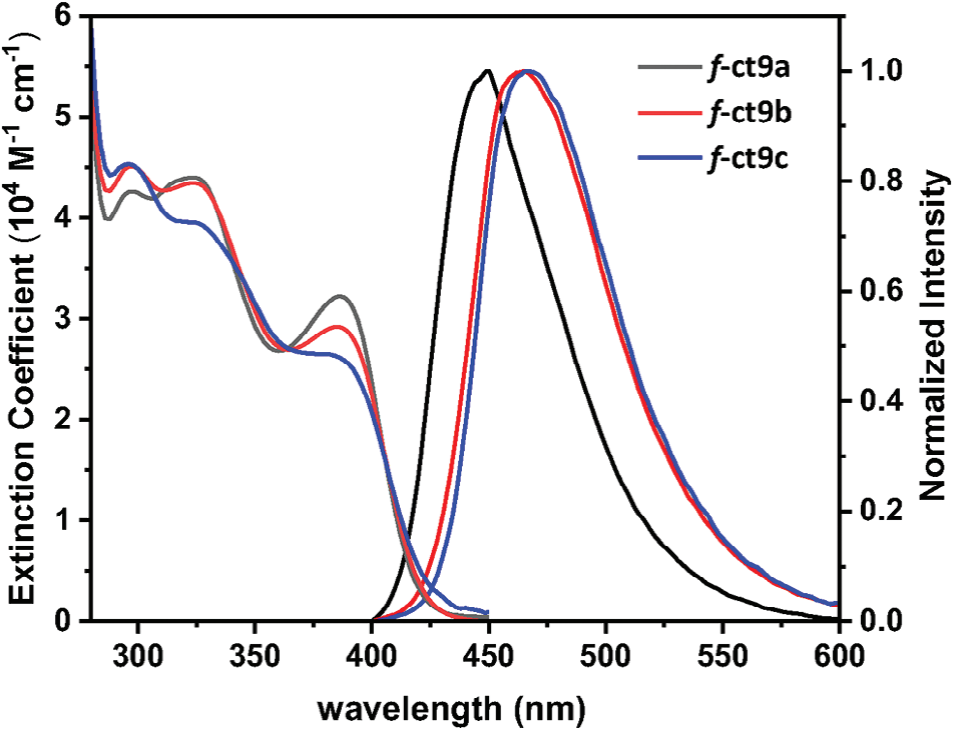
Absorption and emission spectra of studied Ir(III) complexes with a concentration of 10^−5^ M in toluene at RT.

UV–vis absorption and photoluminescence (PL) spectra were recorded in toluene and degassed toluene solution respectively. Notably, these Ir(III) complexes exhibited two sets of absorption bands at regions *λ*_abs_ < 350 nm and *λ*_abs_ ≈386 nm, respectively (**Figure** **1** and **Table** **1**), to which the higher energy bands were assigned to the mixed ligand‐to‐ligand charge transfer (LLCT) and ligand‐centered π–π* transitions, while the lower energy absorption was attributed to the spin allowed metal‐to‐ligand charge transfer (MLCT) transition process. The relatively high extinction coefficient of the lower energy band at ≈386 nm (ε > 26000 M^−1^·cm^−1^) is in accordance with the *f*‐arranged Ir(III) complexes, as confirmed by the single crystal X‐ray analyses. Furthermore, their phosphorescent emission occurred at PL *λ*_max_ of 448, 464, and 467 nm for *f*‐ct9a, *f*‐ct9b, and *f*‐ct9c, respectively. The ascending trend is consistent with the reduced transition energy gap caused by the increased electron density at the Ir(III) center. To be specific, this is due to the stepwise replacement of phenyl with *t*‐butylphenyl cyclometalates, to which the electron‐donating *t*‐butyl group is expected to induce a gradual increase of electron density at the Ir(III) metal center, resulting in an observed red‐shifting for both *f*‐ct9b and *f*‐ct9c in comparison to *f*‐ct9a. Moreover, high PL quantum yield (*Φ*_PL_ = 81–88%), fast radiative rate constant (*k*_r_ = 0.85–0.93 × 10^6^ s^−1^), and narrowed full width at half maximum (FWHM = 59–67 nm) were observed for these blue‐emissive Ir(III) complexes. These superior properties make them the ideal blue phosphors for the fabrication of efficient OLED devices.^[^ ^17^ ^]^ Finally, there existed an extensive overlap of the tail of the absorption band with their PL onset, occurring at around 410–420 nm for *f*‐ct9a–c. This behavior may suggest a reduced energy gap between the lowest singlet and triplet states (∆*E*_ST_), which is an important criterion for the existence of TADF contribution in addition to phosphorescence.^[^ ^18^ ^]^ If affirmative, this could explain the shortened radiative lifetimes detected for this class of blue emissive Ir(III) carbene complexes.

**Table 1:** Photophysical data of the studied Ir(III) complexes in toluene at RT.

	λabs max / nm (ε × 10^4^ M^−1^ cm^−1^)	λPL max [nm] ^a)^	FWHM [nm]	*Φ*_PL _[%] ^a)^, ^b)^	*τ*_obs_ [µs] ^a)^	*τ*_rad_ [µs]	*k*_r_ [10^6^ s^‒1^]	*k*_nr_ [10^5^ s^−1^]
*f*‐ct9a	298 (4.26), 322 (4.01), 386 (3.22)	448	59	81	0.87	1.07	0.93	2.18
*f*‐ct9b	298 (4.51), 324 (4.34), 386 (2.92)	464	67	88	1.04	1.18	0.85	1.15
*f*‐ct9c	296 (4.54), 326 (3.94), 386 (2.62)	467	66	88	1.00	1.14	0.88	1.20

^a)^ A solution of 10^−5^ M was degassed before the measurement;

^b)^ Coumarin 102 in methanol (Q.Y. = 87%, *λ*_max_ = 480 nm) was employed as standard.

### 2.3 Electrochemical and Thermal Properties

Their electrochemical potentials were examined using cyclic voltammetry (CV) (**Table** **2**, and Figure S5, Supporting Information). From the obtained onset oxidation potentials, their HOMO energy levels were estimated to be −5.46, −5.44, and −5.42 eV for *f*‐ct9a–c, the respective HOMO energy levels are consistent with the density functional theory (DFT) predictions as shown (vide infra). Their emission onsets were next employed for the calculation of their optical energy gaps. After that, their LUMO energy levels were estimated using the equations as stated in the footnotes. Furthermore, all Ir(III) complexes were subjected to thermogravimetric analysis (Table 2, and Figure S6, Supporting Information), from which the decomposition temperature (*T_d_*) of 5% weight loss was recorded to be 457, 460, and 469 °C, which are higher than those of other Ir(III) carbene complexes without carbonitrile substituent. Therefore, this class of carbene complexes was highly stable and could withstand the thermal heating exerted during vacuum vapor deposition.

**Table 2:** Electrochemical and thermogravimetric data of the studied Ir(III) complexes.

Complex	*E*ox onset [eV]^a)^	*E*_HOMO_ [eV]^b)^	*E*opt g [eV]^c)^	*E*_LUMO_ [eV]^d)^	*T*_d, 5%_ [°C]^e)^
*f*‐ct9a	0.66	−5.46	3.00	−2.46	457
*f*‐ct9b	0.64	−5.44	2.91	−2.53	460
*f*‐ct9c	0.62	−5.42	2.88	−2.54	469

^a)^ Electrochemical potentials were measured in a 0.1 M DCM solution of TBAPF_6_ referenced to the Fc/Fc^+^ couple; *E*ox onset is the onset potential for the oxidation wave;

^b)^ HOMO = ‐(*E*ox onset + 4.8);

^c)^ energy gap = 1240 / [PL_onset_ (nm)];

^d)^ LUMO = HOMO + energy gap;

^e)^ TGA is recorded under N_2_ flow.

### 2.4 Theoretical Investigation

We investigated the lowest singlet (S_1_) and triplet (T_1_) excited states of Ir(III) complexes *f*‐ct9a–c using the time‐dependent (TD)‐DFT methods.^[^ ^19^ ^]^ Our goal was to understand the variation of spectral bands in response to the chelate modification. Computational details were depicted in the Supporting Information. The vertical excitation energies of the S_0_ → S_1_ transition are calculated to be 412, 417, and 422 nm for *f*‐ct9a–c, respectively (**Table** **3**). These values align with the experimental absorption tails at ≈400 nm (Figure 1). For the S_0_ → T_1_ transition, the vertical excitation energies are calculated to be 440, 445, and 448 nm for ***f*‐ct9a–c**, respectively (Table 3). The latter deviates (with a mean absolute deviation (MAD) of ≈0.09 eV (2.1 kcal mol^−1^)) slightly from the observed phosphorescence peak max. (λPL max) at 448, 464, and 467 nm, respectively (Figure 1 and Table 1).

**Table 3:** The calculated lowest singlet (S_1_) and triplet (T_1_) excited states, main orbital contributions of the S_0_ → S_1_ / T_1_ transitions, and the assignment of the charge character of S_0_ → T_1_ excitation at their geometries optimized ground state (S_0_).

	*E*_HOMO_ ^a)^ [eV]	H‐L gap ^a)^ [eV]	Excitation	*λ* ^b)^ [nm/eV]	*f* ^b)^	MO contribution (> 15%) ^b)^	Assignment^c)^					Sum contribution^d)^
							MLCT	ILCT	LC	LMCT	MC	
*f*‐ct9a	−5.68	3.54	S_0_ → T_1_	440/2.82	0	HOMO → LUMO (79.7%)	29.8%	44.5%	22.3%	2.4%	1.1%	94.2%
			S_0_ → S_1_	412/3.01	0.0944	HOMO → LUMO+1 (93.5%)						
*f*‐ct9b	−5.68	3.54	S_0_ → T_1_	445/2.79	0	HOMO → LUMO+1 (42.9%) HOMO → LUMO (30.6%)	27.6%	21.5%	46.6%	3.0%	1.2%	92.7%
			S_0_ → S_1_	417/2.97	0.0526	HOMO → LUMO+1 (90.1%)						
*f*‐ct9c	−5.69	3.51	S_0_ → T_1_	448/2.77	0	HOMO → LUMO (70.9%) HOMO−1 → LUMO (15.7%)	27.8%	22.9%	45.0%	3.0%	1.2%	92.7%
			S_0_ → S_1_	422/2.94	0.0274	HOMO → LUMO (89.7%)						

^a)^ The *E*_HOMO_ and HOMO‐LUMO (H‐L) gap are computed at optimized S_0_ structures at the B3LYP‐D3(BJ)/def2‐SVP level with polarizable continuum model (PCM) for modelling the toluene solvent;

^b)^ The vertical excitation energy (λ), oscillator strength (*f*) and MO contribution were calculated by TD‐DFT using B3LYP functional with PCM for toluene;

^c)^ The percentage was calculated using the IFCT (Hirshfeld) method;

^d)^ The sum contribution is: MLCT + ILCT + LC – LMCT.

**Figure 2:**
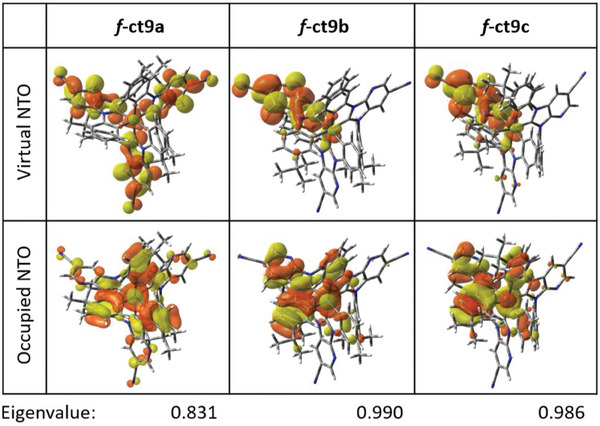
Natural transition orbital (NTO) pairs expressing the S_0_ → T_1_ excitation of *f*‐ct9a–c at their optimized S_0_ geometry, with the dominated NTO eigenvalues.

To gain further insight into their emission properties, we conducted the natural transition orbitals (NTOs) analysis on the S_0_ → T_1_ transitions.^[^ ^20^ ^]^ As shown in **Figure** **2**, the predominant NTO pairs possess a π–π* transition, with the occupied NTOs delocalized on both the Ir(III) center and aryl cyclometalates, while the virtual NTOs are primarily localized at the imidazo[4,5‐b]pyridin‐2‐ylidene fragment.

To evaluate the variation of MLCT, intra‐ligand charge transfer (ILCT), ligand‐centered (LC), ligand‐to‐metal charge transfer (LMCT), and metal‐centered (MC) contributions in response to the S_0_ → T_1_ transition in *f*‐ct9a–c, we employed the inter‐fragment charge transfer (IFCT) method from the Multiwfn software package.^[^ ^21^ ^]^ The calculated MLCT characters are 29.8%, 27.6%, and 27.8% for *f*‐ct9a–c (Table 3), suggesting the reduced dependence of radiative decay rate (*k*_r_) versus the number of 4‐*t*‐butylphenyl cyclometalates. The calculated “net MLCT” (i.e., MLCT–LMCT, 27.4% to 24.5% and 24.8% for *f*‐ct9a–c, respectively) are in good agreement with the measured *k*_r_ values (in µs^−1^, Table 1) of 0.93 (*f*‐ct9a), 0.85 (*f*‐ct9b), and 0.88 (*f*‐ct9c) in toluene. Moreover, the IFCT method revealed that the S_0_ → T_1_ transition of *f*‐ct9a–c has mixed MLCT, ILCT, and LC characters, with contributions of 29.8%, 44.5%, and 22.3% for *f*‐ct9a; 27.6%, 21.5%, and 46.6% for *f*‐ct9b; 27.8%, 22.9%, and 45.0% for *f*‐ct9c (Table 3). The sum contribution of “MLCT+ILCT+LC–LMCT” for each complex was 94.2%, 92.7%, and 92.7%, respectively, further supporting the idea that *f*‐ct9a should have the fastest radiative rate constant (*k*_r_ = 0.93 µs^−1^, Table 1), while *f*‐ct9b and *f*‐ct9c have relatively smaller rate constant (0.85 and 0.88 µs^−1^, respectively, Table 1).

Based on the IFCT predictions, we utilized the spin‐orbit coupling (SOC)‐TDDFT method to calculate the *τ*_rad_ and *k*_r_ data at their optimized triplet (T_1_) and the ground state (S_0_) structure. Previous works have indicated that the τ_rad_ can be determined using either the optimized S_0_ or T_1_ structures.^[^ ^18^, ^22^ ^]^ It has been suggested that the “true” emitting species lies between the S_0_ and T_1_ structures.^[^ ^23^ ^]^ Their arithmetic averages (**Table** **4**) are adapted in the subsequent discussion for simplicity. For the T_1_ → S_0_ emission of *f*‐ct9a, both the predicted *τ*_rad_ of 1.14 µs (at the S_0_ structure) and 0.93 µs (at the T_1_ structure) are nearly identical to the experimental *τ*_rad_ (1.04 µs, Table 1). For *f*‐ct9b and *f*‐ct9c, the experimental τ_rad_ (1.18 µs) and (1.14 µs) are also found in between the predicted values of 0.83 µs (at the S_0_ structure) and 1.53 µs (at the T_1_ structure) and the *τ*_rad_ values of 0.82 µs (at the S_0_ structure) and 1.39 µs (at the T_1_ structure), respectively. Given the relatively large uncertainty (≈1.7 µs) in the theoretical predictions of τ_rad_ value for other Ir(III) complexes by the SOC‐TDDFT method,^[^ ^24^ ^]^ it is suggested that the “actual” triplet emitting species of these Ir(III) complexes (*f*‐ct9a–c) have an initial structure between the S_0_ and T_1_ structures but likely being closer to the T_1_ states.

**Table 4:** The calculated adiabatic and vertical emission energy of T_1_ → S_0_ transition, the emission radiative lifetime (*τ*_rad_), and radiative rate (*k*_r_) of *f*‐ct9a–c.

Emission (T_1_ → S_0_)	*λ* ^a)^ [nm/eV]	*λ* ^b)^ [nm/eV]	*τ*_rad_ ^c)^ [µs]	*k*_r_ ^c)^ [µs^−1^]
*f*‐ct9a	435/2.85	446/2.78 (485/2.56)	1.14/1.16 (0.93/0.98)	0.88/0.86 (1.08/1.02)
*f*‐ct9b	451/2.75	450/2.76 (540/2.30)	0.83/0.97 (1.53/1.71)	1.21/1.03 (0.65/0.58)
*f*‐ct9c	456/2.72	452/2.74 (558/2.22)	0.82/0.96 (1.39/1.57)	1.23/1.04 (0.72/0.64)

^a)^ The adiabatic emission energy obtained from the optimized structures of T_1_ and S_0_ states at B3LYP‐D3(BJ)/def2‐SVP level with PCM for toluene;

^b)^ The vertical SOC‐TDDFT emission energy between T_1_ and S_0_ states at optimized S_0_ structure (in normal font) and T_1_ structure (in italic and bold font in parentheses);

^c)^ The τ_rad_ and *k*_r_ are calculated by the arithmetic/Boltzmann average (at 298 K) of the SOC substates of T_1_, at optimized S_0_ structure (in normal font) and T_1_ structure (in italic and bold font in parentheses).

### 2.5 Device Fabrication and Characterization

**Figure 3:**
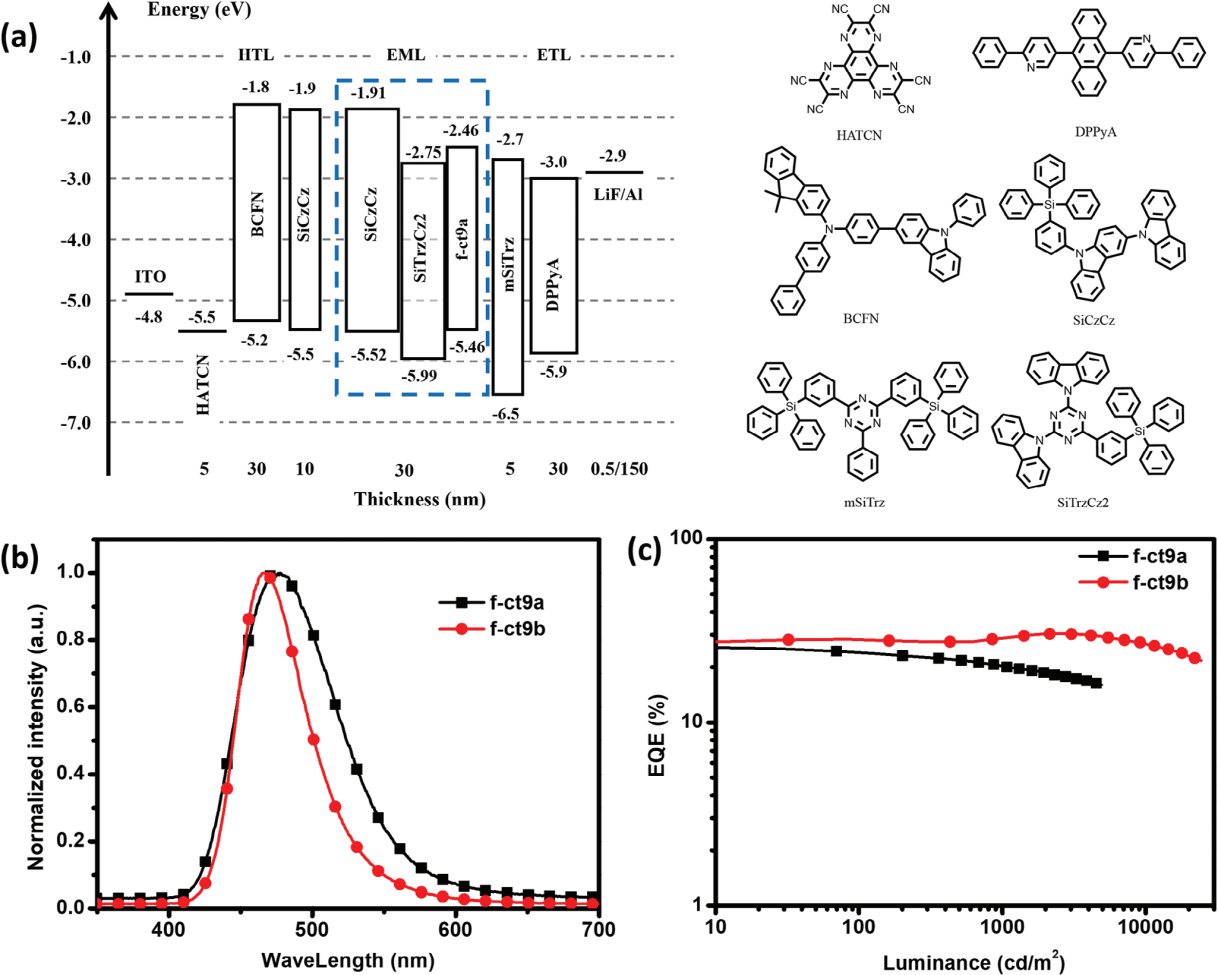
a) the structure of device I, and energy level diagram and chemical structures of the materials employed; b) EL spectra of devices; c) external quantum efficiency versus luminance characteristic of devices.

Owing to the high synthetic yields and impressive thermal stabilities and photophysical properties, *f*‐ct9a and *f*‐ct9b were employed as dopant emitters in the fabrication of OLEDs using the following device architecture (device I): ITO/1,4,5,8,9,11‐hexaazatriphenylenehexcarbonitrile (HATCN, 5 nm)/ [N‐([1,1’‐biphenyl]‐4‐yl)‐9,9‐dimethyl‐N‐(4‐(9‐phenyl‐9Hcarbazol‐3‐yl)phenyl)‐9H‐fluoren‐2‐amine (BCFN, 30 nm)/ 9‐(3‐(triphenylsilyl)phenyl)‐9H‐3,9’‐bicarbazole (SiCzCz, 10 nm)/ 65 wt% SiCzCz: 35 wt% SiTrzCz2: *x* wt% f‐ct9a or f‐ct9b (30 nm)/ 2‐phenyl‐4,6‐bis(3‐(triphenylsilyl)phenyl)‐1,3,5‐triazine (mSiTrz, 5 nm)/ 9,10‐bis(6‐phenylpyridin‐3‐yl)anthracene (DPPyA, 30 nm)/ LiF (0.5 nm)/Al (150 nm). For the emitting layer (EML), the exciplex‐forming host, that is, SiCzCz and SiTrzCz2 (9,9’‐(6‐(3‐(triphenylsilyl)phenyl)‐1,3,5‐triazine‐2,4‐diyl)‐bis(9H‐carbazole)) in a ratio of 0.65:0.35,^[^ ^25^ ^]^ were co‐evaporated with either *f*‐ct9a or *f*‐ct9b, to which the concentrations (*x* wt%) of dopants was varied from 10 to 25 wt% in seeking the optimal device performances. The energy level diagram and chemical structures of the employed materials used in this set of devices are shown in **Figure** **3**a.

As depicted in Figure S7, Supporting Information, and **Table** **5**, all fabricated OLED devices exhibited low turn‐on voltages (2.6–2.8 V), which might be facilitated by the close‐lying HOMO levels between the host and dopants, which is known for inducing better hole transporting character in the EML.^[^ ^26^ ^]^ Furthermore, the low‐lying LUMOs of the dopants stabilized by the cyano substituents also enable balanced carrier (both electron and hole) transport within the devices. Notably, all devices showed higher current density and, particularly, superior performances were observed at a doping ratio of 20 wt%, to which the electroluminescence (EL) data and detailed metric parameters are depicted in Figure 3 and Table 5, respectively.

**Table 5:** Electroluminescence data of devices (device I).

Devices		V [V]	EL [nm]	FWHM [nm]	CE [cd·A^−1^]	PE [lm·W^−1^]	EQE [%] (max / 1000 / 5000 cd·m^−2^)	CIE [x,y] @ 1000 cd·m^−2^
*f*‐ct9a conc.	10%	2.6	472	80	38.1	44.4	23.9 / 17.8 / 15.1	0.150, 0.220
	15%	2.6	473	79	38.5	44.8	23.8 / 19.4 / 16.8	0.151, 0.231
	20%	2.6	478	81	51.9	60.4	25.9 / 20.0 / 16.1	0.157, 0.255
	25%	2.6	475	75	38.3	44.5	22.7 / 18.0 / 15.5	0.149, 0.230
*f*‐ct9b conc.	10%	2.8	468	63	34.4	37.3	23.6 / 18.3 / 14.2	0.152, 0.196
	15%	2.8	466	57	33.7	34.2	25.6 / 21.7 / 17.8	0.143, 0.167
	20%	2.8	468	57	39.5	39.2	30.3 / 29.0 / 29.0	0.142, 0.169
	25%	2.8	466	57	40.0	41.9	29.9 / 28.3 / 26.0	0.142, 0.173

As can be seen in Figure 3, the EL spectrum of *f*‐ct9b‐based devices was akin to its photoluminescence recorded in toluene solution and with peaking (*λ*_max_) at 468 nm, which delivered an maximum external quantum efficiencies (EQE_max_) of 30.3%, current efficiency (CE) of 39.5 cd·A^−1^, power efficiency (PE) of 39.2 lm·W^−1^, and Commission Internationale de L'Eclairage (CIE_(x,y)_) coordinates of (0.142, 0.169). Moreover, the EQE remained to be ≈29.0% at 5000 cd·m^−2^, which is even higher than that reported by its pyrazinyl predecessor *f*‐ct1c (12.8%),^[^ ^27^ ^]^ confirming the balanced carrier transport. Thirdly, there is also a negligible EL spectrum broadening for *f*‐ct9b in comparison to that of *f*‐ct9a, plus an extremely small efficiency roll‐off, that is, EQE of 26.6% at 10 000 cd·m^−2^, showing the reduced triplet‐triplet annihilation (TTA) and triplet‐polaron annihilation (TPA) possibly induced by its multiple *tert*‐butyl groups,^[^ ^27^ ^]^ and the cyano substituents that facilitated the electron transport.^[^ ^6^, ^15^, ^28^ ^]^ In contrast, the *f*‐ct9a‐based device exhibited a more broadened spectrum in reference to *f*‐ct9b, giving a slightly inferior EQE_max_ of 25.9%, CE of 51.9 cd·A^‒1^, PE of 60.4 lm·W^−1^, and CIE_(x,y)_ coordinates of (0.157, 0.255). Again, this OLED device still exhibited a high EQE of 16% at 5000 cd·m^−2^, to which the small efficiency roll‐off is uncommon to the blue OLEDs documented in the literature. Notably, the OLED devices based on this series of complexes showed advancement in several aspects (e.g., EQEs) compared with other representative blue OLEDs shown in Table S3, Supporting Information.: *mer*‐Ir(pmp)_3_: TSPO1 (EL_max_ = 465 nm, EQE_max_ = 14.4%, EQE_1000_ = 13.3%),^[^ ^6^ ^]^ *f*‐tpb1: mCBP (EL_max_ = 472 nm, EQE_max_ = 13.5%, EQE_1000_ = 12.0%),^[^ ^6^ ^]^ Ir(cb)_3_: TSPO1 (EL_max_ = 468 nm, EQE_max_ = 17.1%, EQE_200_ = 13.5%).^[^ ^7^ ^]^ Overall, the improvement of EQEs at both maximum and practical brightness of the *f*‐ct9b‐based devices can be ascribed to the strengthened Ir‐carbon bonds of carbene cyclometalates and, hence, better stability.

To further probe their fundamentals, the photoluminescence (PL) properties of both *f*‐ct9a and *f*‐ct9b doped in this exciplex co‐host system were examined. As shown in Table S1 and Figure S8, Supporting Information, both thin films displayed blue emission with high PL quantum yields (*Φ*_PL_ = 71–77%) and fast radiative rate constants (*k*_r_ = 0.74–1.09 × 10^6^ s^−1^). These characteristics confirmed the superior efficiencies obtained in device I. Furthermore, they exhibited narrowed PL with λ_max_ at 468 and 476 nm and FWHM = 63 and 65 nm, slightly different from those observed in their EL spectra (478 and 468 nm and FWHM = 63 and 65 nm). These variations were tentatively attributed to the low electron mobility of the mixed co‐host.

To provide further evidence, we attempted the fabrication of a second set of OLED devices employing 2,8‐bis(diphenylphosphoryl)dibenzo[b,d]furan (PPF) as the high triplet energy and better electron transporting host material (device II, Figure S9, Supporting Information), to which the performance characteristics were depicted in Figure S10 and Table S2, Supporting Information for scrutiny. As expected, *f*‐ct9a‐based device delivered a narrowed emission profile, and a maximum efficiency of 28.8% at a doping concentration of 15 wt%, showing the potential of *f*‐ct9a for high‐performance OLED devices. However, their relative performances, that is, *f*‐ct9a versus *f*‐ct9b, are in reverse to those obtained in the sets of device I.

**Figure 4:**
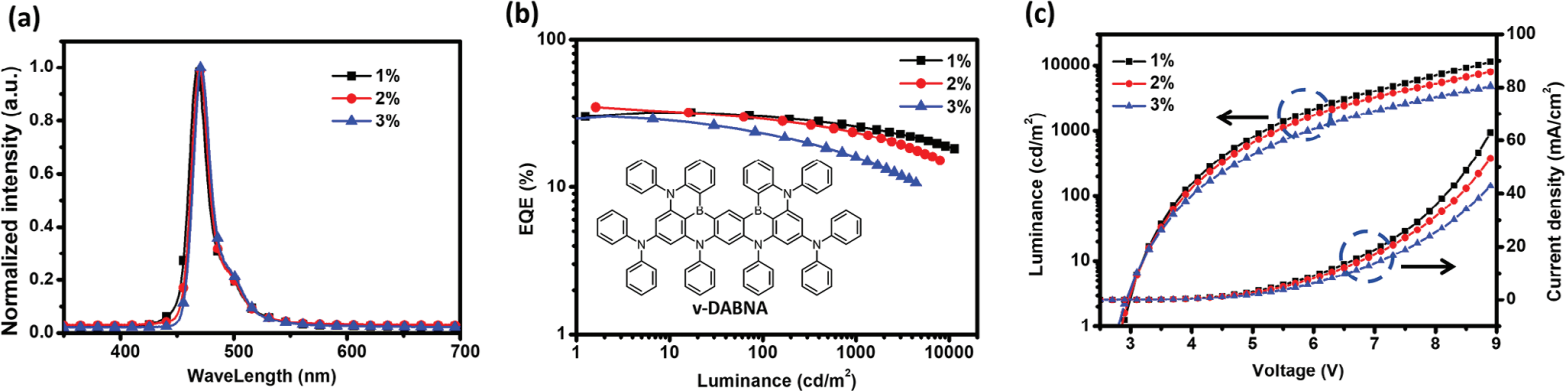
a) EL spectra, b) EQE versus luminance, and c) *J–V–L* curves of *f*‐ct9b based hyper‐OLEDs.

Recently, hyper‐OLED (or hyperphosphorescence) has gained research momentum in the fabrication of efficient OLED devices.^[^ ^29^ ^]^ This seminal design was first documented by Baldo and co‐workers utilizing an efficient Förster resonance energy transfer (FRET) process using multi‐layer EML.^[^ ^30^ ^]^ It next triggered a series of studies on the phosphor sensitized fluorescent,^[^ ^31^ ^]^ and both the TADF and phosphor‐sensitized TADF devices.^[^ ^32^ ^]^ Still, the Ir(III) carbene complexes should be one of the best dopant sensitizers due to their high stability, better emission efficiency, and short radiative lifetime.^[^ ^33^ ^]^ Encourage by the aforementioned precedents, hyperphosphorescence was initiated from *f*‐ct9b to the narrowband multi‐resonance MR‐TADF terminal emitter 5H,9H,11H,15H‐[1,4] benzazaborino[2,3,4‐kl][1,4]benzazaborino[4’,3’,2’:4,5][1,4]benzazaborino[3,2‐b]phenazaborine‐7,13‐diamine, N7,N7,N13,N13,5,9,11,15‐octaphenyl (ν‐DABNA). These hyper‐OLED devices (device III) with configuration of ITO / HATCN (5 nm) / BCFN (30 nm) / SiCzCz (10 nm) / 65 wt% SiCzCz: 35 wt% SiTrzCz2: 20 wt% *f*‐ct9b: *x* wt% ν‐DABNA (30 nm) / mSiTrz (5 nm) / DPPyA (30 nm) / LiF (0.5 nm) / Al (150 nm) were constructed. As depicted in **Figure** **4** and **Table** **6**, the low torn‐on voltages were maintained, as the employed concentration of ν‐DABNA (1–3 wt%) would not seriously affect the charge transporting properties. Moreover, all these devices exhibited the expected narrowband emission with full width at half maximum (FWHM) of 18–19 nm, indicating an efficient FRET process. Remarkably, the champion device was obtained using 2 wt% ν‐DABNA, delivering a blue emission centered at 469 nm and an EQE_max_ of 34.7% with CIE_(x,y)_ coordinates of (0.122, 0.131). These performances confirmed that these cyano‐modified Ir(III) carbene phosphors are also capable of serving as the phosphor sensitizer in achieving high‐efficiency blue OLEDs.

**Table 6:** Electroluminescence data of devices (device III).

Devices		V [V]	EL [nm]	FWHM [nm]	CE [cd·A^−1^]	PE [lm·W^−1^]	EQE [%] (max / 1000 / 5000 cd·m^−2^)	CIE [x,y] @ 1000 cd·m^−2^
v‐DABNA conc.[%]								
*f*‐ct9b	1%	2.9	468	19	31.0	31.5	32.1 / 25.5 / 21.3	0.127, 0.123
	2%	2.9	469	18	32.2	34.8	34.7 / 23.0 / 17.1	0.122, 0.131
	3%	2.9	471	18	34.6	37.5	30.1 / 15.4 / 10.4	0.123, 0.153

Finally, the operational lifetimes of device I were estimated at an initial luminance of 1000 cd m^−2^. As shown in Figure S11, Supporting Information, the devices using *f*‐ct9b exhibited longer LT_50_ than those of its counterpart *f*‐ct9a under our employed conditions, and the result was consistent with the higher maximum luminance of *f*‐ct9b‐based devices, which were attributed to the relatively slow non‐radiative rate constant.

## 3 Summary and Conclusion

To conclude, we prepared a new series of Ir(III) carbene complexes (***f*‐ct9a–c**), whose structures were examined by single crystal X‐ray diffraction studies and their photophysical properties were comprehensively investigated. The high PLQYs (*Φ*_PL_ = 81–88%) of these Ir(III) complexes confirmed the successfulness of employing cyano‐modified imidazo[4,5‐b]pyridin‐2‐ylidene chelates, revealing their potential in OLED applications. Hence, *f*‐ct9a and *f*‐ct9b were employed as dopants in the fabrication of OLEDs, which delivered impressive EQE_max_ of 25.9% and 30.3%, respectively. Notably, the *f*‐ct9b‐based device presented extremely small efficiency roll‐offs (EQEs of 29.0% and 26.6% at high brightness of 5000 and 10 000 cd·m^−2^, respectively), together with a maximum luminance of 24 400 cd·m^‒2^ at the current density of 88 mA cm^−2^. Additionally, the corresponding hyper‐OLED with *f*‐ct9b as the phosphorescent dopant sensitizer and v‐DABNA as the terminal emitter, delivered a narrowband blue emission with CIE_x,y_ coordinates of (0.122, 0.131), and a high EQE_max_ of 34.7%. These results demonstrated that the cyano‐substituted imidazo[4,5‐b]pyridin‐2‐ylidene‐based Ir(III) complexes are indeed suitable candidates in constructing highly efficient blue OLEDs for practical applications in the future.

## 4 Experimental Section

### General Information and Materials

All chemical reagents and deuterated solvents were purchased from commercial suppliers and used directly. All reactions were conducted under an inert atmosphere. Bruker Avance III NMR instrument (400 MHz) was employed to record the respective ^1^H and ^19^F NMR spectra. A high‐resolution mass spectrometer (Sciex X500R Q‐TOF) was used to obtain the HRMS data. TGA measurements were performed on a thermogravimetric analyzer TGAQ50 at a heating rate of 10 °C min^−1^ under N_2_ atmosphere.

### Synthesis of Ir(III) Complexes *f*‐ct9a, *f*‐ct9b and *f*‐ct9c

Chelate H4 (1.22 g, 2.4 mmol), sodium acetate (2.04 g, 24 mmol), *mer‐*IrCl_3_(tht)_3_ (450 mg, 0.8 mmol), and *o‐*dichlorobenzene (40 mL) were added to a 100 mL flask. The mixture was refluxed for 24 h with vigorous stirring. After the removal of solvent under vacuum, the residue was taken into CH_2_Cl_2_ and washed with deionized water. The organic phase was separated, dried over anhydrous Na_2_SO_4_, and concentrated. The residue was purified by column chromatography with a mixture of *n*‐hexane, ethyl acetate, and CH_2_Cl_2_ (8/3/2, v/v/v) to afford *f*‐ct9a (*R_f_* = 0.7), *f*‐ct9b (*R_f_* = 0.6), and *f*‐ct9c (*R_f_* = 0.3) in sequence. Further recrystallization with CH_2_Cl_2_ (or CHCl_3_) and methanol attained a yellow solid of *f*‐ct9a (289 mg, 29%), *f*‐ct9b (123 mg, 22%), and *f*‐ct9c (40 mg, 4.0%), respectively.

### Spectroscopic Data of f‐ct9a

HRMS (ESI) for C_69_H_58_IrN_12_ [M]^+^: calcd 1247.4531, found 1247.4496; ^1^H NMR (400 MHz, CDCl_3_) *δ* 8.90 (d, *J* = 7.6 Hz, 3H), 8.67 (d, *J* = 1.6 Hz, 3H), 7.41 (dd, *J* = 8.3, 1.9 Hz, 3H), 7.19 (t, *J* = 7.6 Hz, 3H), 6.89 (d, *J* = 1.6 Hz, 3H), 6.84 (t, *J* = 7.2 Hz, 3H), 6.58 (d, *J* = 7.2 Hz, 3H), 6.43 (dd, *J* = 8.3, 1.9 Hz, 3H), 6.29 (dd, *J* = 8.3, 2.3 Hz, 3H), 6.22 (dd, *J* = 8.3, 2.3 Hz, 3H), 1.06 (s, 27H). Anal. Calcd. for C_69_H_57_IrN_12_: C, 66.49; H, 4.61; N, 13.48. Found: C, 66.51; H, 4.62; N, 13.50.

### Selected Crystal Data of f‐ct9a

CCDC number: 2250716. C_72.5_H_60.5_Cl_10.5_IrN_12_; *M* = 1664.25; monoclinic; space group P2_1_/n; *a* = 19.4901(4) Å, *b* = 14.8227(3) Å, *c* = 31.1525(7) Å; *β* = 105.3920(10)°; *V* = 8677.0(3) Å^3^; *Z* = 4; ρ_Calcd_ = 1.274 g·cm^−3^; *µ* = 6.295 mm^−1^; F(000) = 3340, *λ*(Cu‐K_α_) = 1.54178 Å; *T* = 213 (2) K; index range: −24 ≤ *h* ≤ 24, −15 ≤ *k* ≤ 18, −38 ≤ *l* ≤ 38; 109 670 reflections collected, 17 750 independent reflections (*R_int_* = 0.0621), data/restraints/parameters = 17 750/120/922, GOF = 1.058, final R_1_[*I* > 2σ(*I*)] = 0.0481 and *w*R_2_(all data) = 0.0525.

### Spectroscopic Data of f‐ct9b

HRMS (ESI) for C_69_H_58_IrN_12_ [M]^+^: calcd 1247.4531, found 1247.4494; ^1^H NMR (400 MHz, CDCl_3_) *δ* 8.95 (d, *J* = 7.9 Hz, 1H), 8.82 (d, *J* = 7.9 Hz, 1H), 8.69 (d, *J* = 1.7 Hz, 1H), 8.64 (d, *J* = 1.7 Hz, 1H), 8.58 (d, *J* = 1.4 Hz, 1H), 8.34 (d, *J* = 1.4 Hz, 1H), 7.63 (d, *J* = 8.2 Hz, 1H), 7.52 (dd, *J* = 8.2, 2.0 Hz, 1H), 7.41 (dd, *J* = 8.2, 2.0 Hz, 1H), 7.26 – 7.11 (m, 5H), 7.07 (d, *J* = 1.7 Hz, 1H), 6.96 (d, *J* = 1.7 Hz, 1H), 6.91–6.75 (m, 4H), 6.66 (d, *J* = 7.3 Hz, 1H), 6.61 (d, *J* = 2.0 Hz, 1H), 6.50 (d, *J* = 6.5 Hz, 2H), 6.40 (dd, *J* = 8.4, 2.0 Hz, 1H), 6.37–6.30 (m, 2H), 6.24 (dd, *J* = 8.4, 2.0 Hz, 2H), 6.21–6.06 (m, 2H), 1.04 (s, 9H), 1.01 (s, 9H), 1.00 (s, 9H). Anal. Calcd. for C_69_H_57_IrN_12_: C, 66.49; H, 4.61; N, 13.48. Found: C, 66.53; H, 4.60; N, 13.52.

### Selected Crystal Data of f‐ct9b

CCDC number: 2250719. C_70.5_H_59_Cl_4_IrN_12_; M = 1408.29; monoclinic; space group P2/c; *a* = 26.3878(12) Å, *b* = 19.5424(11) Å, *c* = 26.7659(12) Å; *β* = 90.311(4)°; *V* = 13802.5(12) Å^3^; *Z* = 8; ρ_Calcd_ = 1.355 g·cm^−3^; *µ* = 5.553 mm^−1^; F(000) = 5688, *λ*(Cu‐K_α_) = 1.54178 Å; *T* = 213 (2) K; index range: −32 ≤ *h* ≤ 32, −24 ≤ *k* ≤ 18, −33 ≤ *l* ≤ 33; 184 838 reflections collected, 28 235 independent reflections (*R_int_* = 0.0712), data/restraints/parameters = 28 235/574/1696, GOF = 1.048, final R_1_[*I* > 2σ(*I*)] = 0.0400 and *w*R_2_(all data) = 0.1104.

### Spectroscopic data of f‐ct9c

HRMS (ESI) for C_69_H_58_IrN_12_ [M]^+^: calcd 1247.4531, found 1247.4597; ^1^H NMR (400 MHz, CDCl_3_) *δ* 8.88 (d, *J* = 8.0 Hz, 1H), 8.67 (d, *J* = 1.8 Hz, 1H), 8.63 (d, *J* = 1.6 Hz, 1H), 8.47 (d, *J* = 1.6 Hz, 1H), 8.36 (d, *J* = 1.5 Hz, 1H), 8.35 (d, *J* = 1.5 Hz, 1H), 7.71 (d, *J* = 8.3 Hz, 1H), 7.55 (d, *J* = 8.4 Hz, 1H), 7.52 (dd, *J* = 8.9, 2.4 Hz, 1H), 7.4z0–7.32 (m, 2H), 7.22–7.15 (m, 2H), 7.10 (dd, J = 7.7, 2.7 Hz, 1H), 7.11 (d, J = 1.8 Hz, 1H), 6.92–6.81 (m, 3H), 6.76 (s, 1H), 6.68 (d, *J* = 2.2 Hz, 1H), 6.65 (dd, *J* = 8.4, 2.2 Hz, 1H), 6.54 (dd, *J* = 7.4, 1.0 Hz, 1H), 6.46 (d, *J* = 2.2 Hz, 1H), 6.43–6.11 (m, 7H), 1.05 (s, 9H), 1.01 (s, 9H), 0.99 (s, 9H). Anal. Calcd. for C_69_H_57_IrN_12_: C, 66.49; H, 4.61; N, 13.48. Found: C, 66.53; H, 4.60; N, 13.52.

### Selected Crystal Data of f‐ct9c

CCDC number: 2250723. C_72_H_60_Cl_9_IrN_12_; M = 1604.57; monoclinic; space group P2_1_/c; *a* = 12.3106(3) Å, *b* = 24.5073(5) Å, *c* = 23.6068(5) Å; *β* = 8.1730(10)°; *V* = 7049.8(3) Å^3^; *Z* = 4; ρ_Calcd_ = 1.512 g·cm^−3^; *µ* = 2.287 mm^−1^; F(000) = 3224, *λ*(Mo‐K_α_) = 0.71073 Å; *T* = 213 (2) K; index range: −15 ≤ *h* ≤ 15, −30 ≤ *k* ≤ 30, −29 ≤ *l* ≤ 29; 63 913 reflections collected, 14 452 independent reflections (*R_int_* = 0.0676), data/restraints/parameters = 14 452/174/886, GOF = 1.004, final R_1_[*I* > 2σ(*I*)] = 0.0445 and *w*R_2_(all data) = 0.1168.

## Conflict of Interest

The authors declare no conflict of interest.

## Supporting information

Supporting Information

## Data Availability

The data that support the findings of this study are available in the supplementary material of this article.
