# Supplementary material for: Ir(III) Metal Emitters with Cyano‐Modified Imidazo[4,5‐b]pyridin‐2‐ylidene Chelates for Deep‐Blue Organic Light‐Emitting Diodes
Source: Adv Sci (Weinh). 2024 Apr 30;11(26):2309389. doi: 10.1002/advs.202309389 (PMC11234470; doi:10.1002/advs.202309389)
Supplement: Supplementary file 1 — Supporting Information [file ADVS-11-2309389-s001.pdf]

ADVANCED SCIENCE
Open Access

Supporting Information

for *Adv. Sci.*, DOI 10.1002/advs.202309389

Ir(III) Metal Emitters with Cyano-Modified Imidazo[4,5-b]pyridin-2-ylidene Chelates for Deep-Blue Organic Light-Emitting Diodes

Yixin Wu, Yangyang Xin, Yi Pan, Shek-Man Yiu, Jie Yan, Kai Chung Lau*, Lian Duan* and Yun Chi**

Supporting Information

Ir(III) metal emitters with cyano modified Imidazo[4,5-b]pyridin-2-ylidene chelates for deep blue organic light emitting diodes

Yixin Wu,[a,†] Yangyang Xin,[b,†] Yi Pan,[a,†] Shek-Man Yiu,[a] Jie Yan,[a,*] Kai Chung Lau,[a,*] Lian Duan,[b,*] Yun Chi,[a,*]

[†] Y.W., Y.X. and Y.P. contributed equally to this work.

(a) Department of Materials Science and Engineering, Department of Chemistry, and Center of Super-Diamond and Advanced Films (COSDAF), City University of Hong Kong, Hong Kong SAR, E-mail: jyanae@connect.ust.hk, E-mail: kaichung@cityu.edu.hk, and E-mail: yunchi@cityu.edu.hk.

(b) Key Laboratory of Organic Optoelectronics and Molecular Engineering of Ministry of Education, Department of Chemistry, Tsinghua University, Beijing 100084 (P. R. China)

E-mail: duanl@mail.tsinghua.edu.cn

Keywords: (iridium, carbene, blue, organic light emitting diodes, cyclometalate)

General information and materials. All reactions were conducted under N_2 atmosphere. Commercially available reagents were used without further purification and solvents were dried prior to use. ^{1}H and ^{19}F NMR spectra were measured with Bruker Avance III 400 MHz NMR instrument. Mass spectra were recorded on a Dionex 2D-LC (Bruker micrOTOF-Q LC/MS/MS) liquid chromatograph-mass spectrometer system. TGA measurements were performed on a TA Instrument TGAQ50, at a heating rate of 10 °C min^{-1} under a nitrogen atmosphere.

Photophysical measurements: UV-Vis spectra were recorded on a HITACHI UH-4150 spectrophotometer. The steady-state emission spectra were measured with Edinburgh FS 980. Both wavelength-dependent excitation and emission responses of the fluorimeter were calibrated. The lifetime studies were performed by a time-correlated single photon counting system (TCSPC). Spectral grade solvents (Merck) were used as received. To determine the photoluminescence quantum yield in solution, samples were degassed using at least three freeze-pump-thaw cycles. The solution quantum yields are calculated using the standard sample which has a known quantum yield.

Electrochemistry: Cyclic voltammetry was conducted on a CHI621A Electrochemical Analyzer. Ag/Ag^+ (0.01 M $AgNO_3$) electrode was employed as reference electrode. The oxidation and reduction potentials were measured using a glassy carbon working electrode with 0.1 M of NBu_4PF_6 in CH_3CN, respectively. The potentials were referenced externally to the ferrocenium/ferrocene (Fc^+/Fc) couple.

Computational details of theoretical investigations: The geometries, electronic structures, and electronic excitations of the studied Ir(III) complexes were investigated at the B3LYP-D3(BJ)/def2-SVP level[1] with Gaussian 16 set of programs.[2] The solvent effect of toluene was taken account by the polarizable continuum model (PCM).[3] The corresponding ground state (S_0) and lowest triplet state (T_1) geometries were optimized based on the X-ray structural data of ***f*-ct9a** – **c**. A total of 10 low-lying excited states (T_1 ~ T_5 and S_1 ~ S_5) were included in the TD–DFT

calculation[4] based on the optimized S_0 structure. Natural transition orbital (NTO) analysis was applied to obtain a clear and compact orbital representation for the electronic excitation described by a variety of orbital transitions without a single predominant one (e.g., $S_0 \rightarrow T_1$ excitation in this work) at optimized S_0 structure.[5] The IFCT (interfragmentary charge transfer) method analysis in the $S_0 \rightarrow T_1$ excitation is using Multiwfn software.[6] The Hirshfeld method is used to calculate the density in IFCT analysis.[7]

The spin-orbit coupling (SOC)-TDDFT calculation[8] was performed using B3LYP functional with ZORA Hamiltonian[9] (SARC-ZORA-SVP for Ir and ZORA-def2-SVP for other elements) at the optimized S_0 and T_1 structures in ORCA (v5.0.3) software.[10] A total of 100 low-lying excited states (50 for singlet and 50 for triplet) were included in the SOC-TDDFT calculation in toluene with COSMO model.[11] The radiative lifetime (τ_{rad}) and radiative rate (k_r) are calculated by the arithmetic average and Boltzmann average (at 298 K) of the three SOC substates of T_1 excited states.[8]

Device fabrication and characterization：OLEDs were fabricated on the ITO-coated glass substrates with multiple organic layers sandwiched between the transparent bottom indium-tin-oxide (ITO) anode and the top metal cathode using a Trovato 450C system. Before device fabrication, the ITO glass substrates were pre-cleaned carefully. All material layers were deposited by vacuum evaporation in a vacuum chamber with a base pressure of 10^{-4} Pa. The deposition rate of organic layers was kept at 1-2 Å/s. The doping was conducted by co-evaporation from separate evaporation sources with different evaporation rates. The current density, voltage, luminance, external quantum efficiency, electroluminescent spectra and other characteristics were measured with a Keithley 2400 sourcemeter. The EQE measurement system is Hamamatsu C9920-12, which is equipped with Hamamatsu PMA-12 Photonic multichannel analyzer C10027-02 whose longest detection wavelength is 1100 nm. All measurements were repeated three times, among which the median value was chosen as the reported data, and the corresponding errors are generally within ±5%.

Preparation of cyano substituted carbene chelate.

5-Bromo-3-nitro-*N*-phenylpyridin-2-amine (H1)

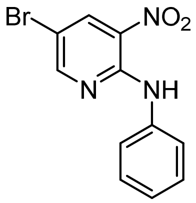

2,5-Dibromo-3-nitropyridine (5.64 g, 20 mmol) was dissolved in a mixed isopropanol/THF solution (100 mL, 4/1, v/v) in a 250 mL flask. Aniline (2.1 g, 22 mmol) and triethylamine (15 g, 100 mmol) were added and, the solution was refluxed with vigorous stirring overnight. After cooled to RT, the mixture was concentrated to dryness and dissolved with ethyl acetate. The solution was washed with distilled water (200 mL × 3) and the organic layer was dried over anhydrous Na_2SO_4, filtered and concentrated to dryness to give a red solid. (**H1**, 5.59 g, 95%).

Spectroscopic data of **H1**: ^{1}H NMR (400 MHz, $CDCl_3$) δ 10.05 (s, 1H), 8.65 (d, J = 2.3 Hz, 1H), 8.50 (d, J = 2.3 Hz, 1H), 7.60 (d, J = 7.7 Hz, 2H), 7.41 (t, J = 7.9 Hz, 2H), 7.21 (t, J = 7.4 Hz, 1H).

6-Bromo-3-phenyl-3*H*-imidazo[4,5-*b*]pyridine (H2)

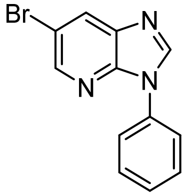

A mixture of 5-bromo-3-nitro-*N*-phenylpyridin-2-amine (4.5 g, 15 mmol) and iron powder (5.3 g, 92 mmol) in formic acid (40 mL) was heated to reflux for 24 hours. After then, formic acid was removed under vacuum and ethyl acetate was added to dissolve the residue. The solution was filtered through Celite and then, washed with distilled water (100 mL), dried over anhydrous Na_2SO_4 and concentrated to dryness. The crude product was further purified via silica gel column chromatography using hexane/ethyl acetate (3/1, v/v) as eluent to afford a white solid (3.8 g, 92%).

Spectroscopic data of **H2**: ^{1}H NMR (400 MHz, $CDCl_3$) δ 8.51 (d, J = 1.9 Hz, 1H), 8.37 (s, 1H), 8.31 (d, J = 2.0 Hz, 1H), 7.72 (d, J = 7.8 Hz, 2H), 7.60 (t, J = 7.8 Hz, 2H), 7.50 (t, J = 7.4 Hz, 1H).

3-Phenyl-3*H*-imidazo[4,5-*b*]pyridine-6-carbonitrile (H3)

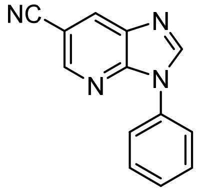

To a 100 mL flask was added 6-bromo-3-phenyl-3*H*-imidazo[4,5-*b*]pyridine (2.74 g, 10 mmol), zinc cyanide (1.41 g, 12 mmol), $Pd(PPh_3)_4$ (0.7 g, 0.6 mmol), and anhydrous DMF (40 mL). The mixture was heated at 120 °C overnight. DMF was removed under reduced pressure, the residue was dissolved in ethyl acetate (60 mL) and washed with a diluted solution of ammonia water (NH_4OH), followed by deionized water. The combined organic phase was dried over anhydrous Na_2SO_4 and concentrated *in vacuo*. The residue was washed with n-hexane to attain white solid (1.82 g, 82%).

Spectroscopic data of **H3**: ^1H NMR (400 MHz, $CDCl_3$) δ 8.72 (s, 1H), 8.48 (s, 1H), 8.45 (s, 1H), 7.71 (d, J = 7.8 Hz, 2H), 7.62 (t, J = 7.8 Hz, 2H), 7.52 (t, J = 7.4 Hz, 1H).

6-Cyano-1-(4-(*tert*-butyl)phenyl)-3-phenyl-3*H*-imidazo[4,5-*b*]pyridin-1-ium trifluoromethane-sulfonate (H4)

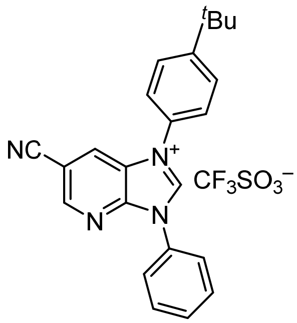

A mixture of 3-phenyl-3*H*-imidazo[4,5-*b*]pyridine-6-carbonitrile (1.76 g, 8 mmol), $(4\text{-}^tBuC_6H_4I^+Mes)(OTf^-)$ (4.64 g, 8.8 mmol) and Cu_2O (57 mg, 0.4 mmol) in anhydrous DMF (20 mL) was heated at 110 °C for 8 h. After cooled to RT, DMF was removed under reduced pressure and the residue was triturated with deionized water (50 mL). The resulting precipitate was filtered, washed with n-hexane, and dried under vacuum to provide a grey solid. It was further purified by recrystallization with acetone and diethyl ether to give white solid (2.6 g, 65%).

Spectroscopic data of **H4**: ^1H NMR (400 MHz, DMSO-d_6) δ 11.00 (s, 1H), 9.33 (s, 1H), 9.32 (s, 1H),

7.98 (d, J = 7.3 Hz, 2H), 7.89 (d, J = 8.7 Hz, 2H), 7.83 (d, J = 8.8 Hz, 2H), 7.80 (t, J = 7.8 Hz, 2H), 7.74 (t, 1H), 1.39 (s, 9H). ^{19}F NMR (376 MHz, DMSO-d_6) δ −77.78 (s, 3F).

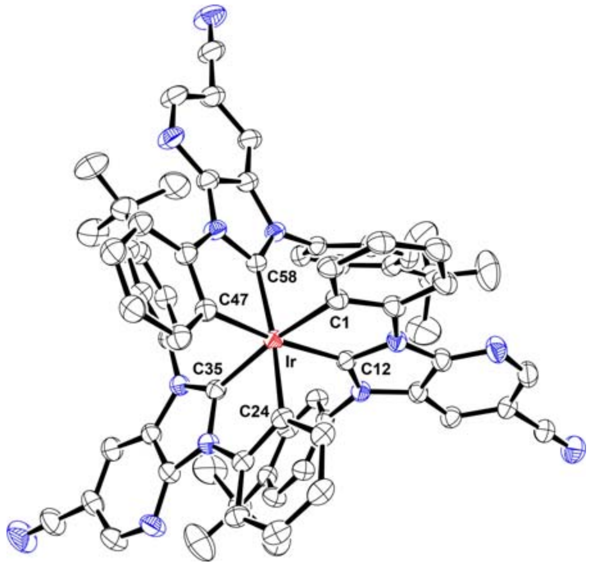

Figure S1. Molecular structure of ***f*-ct9a** with thermal ellipsoids shown at 30% probability level. Selected bond length (Å): Ir-C(12) = 2.032(3), Ir-C(35) = 2.046(3), Ir-C(58) = 2.030(3), Ir-C(1) = 2.099(3), Ir-C(24) = 2.095(4), Ir-C(47) = 2.102(4). Selected bond angle (°): C(12)-Ir-C(1) = 78.64(14), C(58)-Ir-C(47) = 78.51(15), C(35)-Ir-C(24) = 78.25(14).

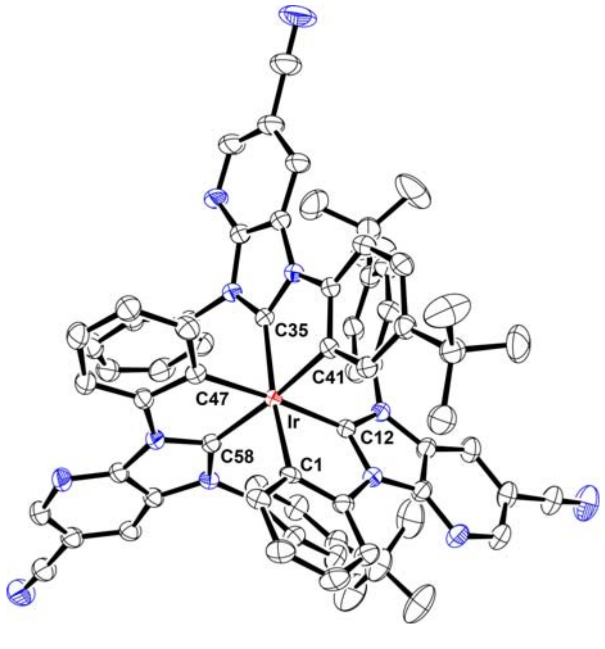

Figure S2. Molecular structure of ***f*-ct9b** with thermal ellipsoids shown at 30% probability level. Selected bond length (Å): Ir-C(12) = 2.018(3), Ir-C(35) = 2.037(3), Ir-C(58) = 2.035(3), Ir-C(1) = 2.092(3), Ir-C(41) = 2.101(3), Ir-C(47) = 2.092(3). Selected bond angle (°): C(47)-Ir-C(58) = 78.25(13),

C(1)-Ir-C(12) = 78.30(13), C(41)-Ir-C(35) = 78.26(14).

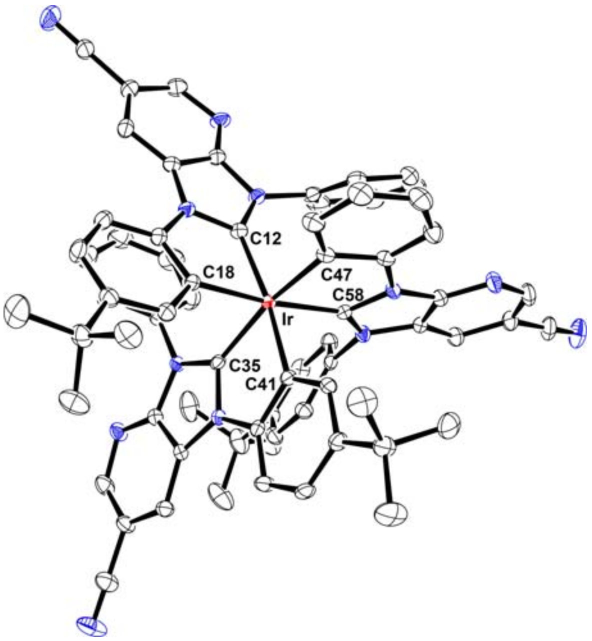

Figure S3. Molecular structure of ***f*-ct9c** with thermal ellipsoids shown at 30% probability level. Selected bond length (Å): Ir-C(12) = 2.036(5), Ir-C(35) = 2.028(5), Ir-C(58) = 2.029(5), Ir-C(18) = 2.095(5), Ir-C(41) = 2.101(5), Ir-C(47) = 2.083(5). Selected bond angle (°): C(18)-Ir-C(12) = 77.77(19), C(41)-Ir-C(35) = 78.04(19), C(47)-Ir-C(58) = 78.39(19).

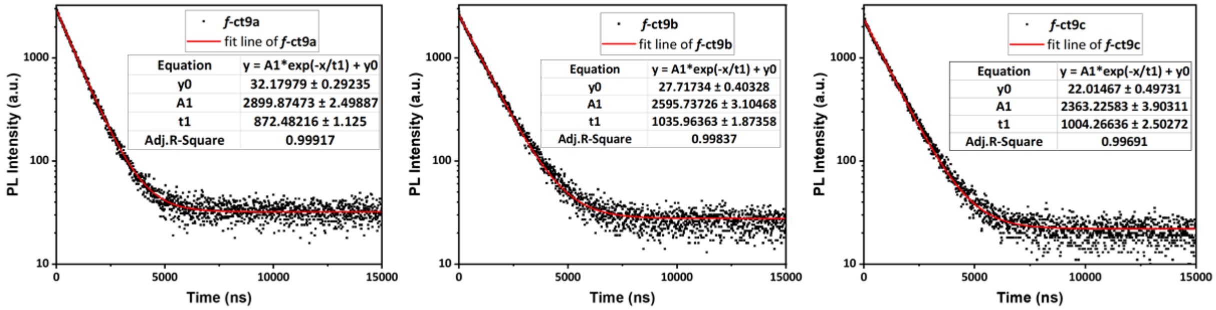

Figure S4. Decay curves and respective best-fit line of ***f*-ct9a** – **c** recorded in 10^{-5} M of degassed toluene at RT.

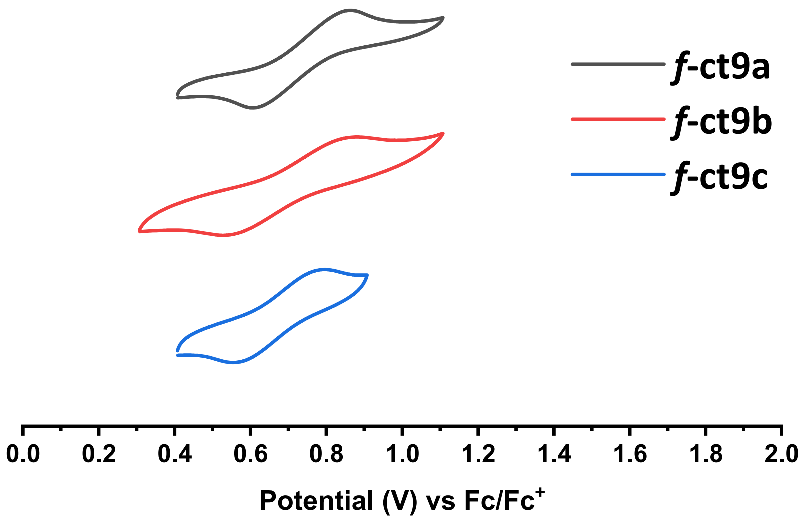

Figure S5. Cyclic voltammograms of ***f*-ct9a** – **c** recorded in CH_2Cl_2 solution.

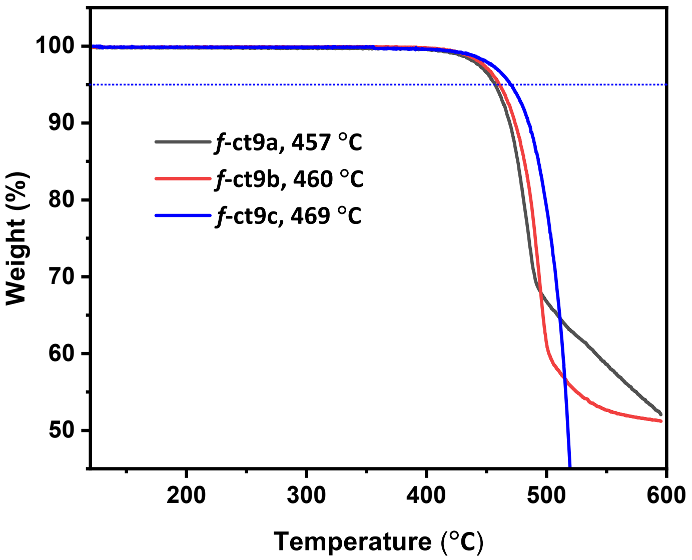

Figure S6. Thermogravimetric diagrams of ***f*-ct9a** – **c** measured under N_2 atmosphere.

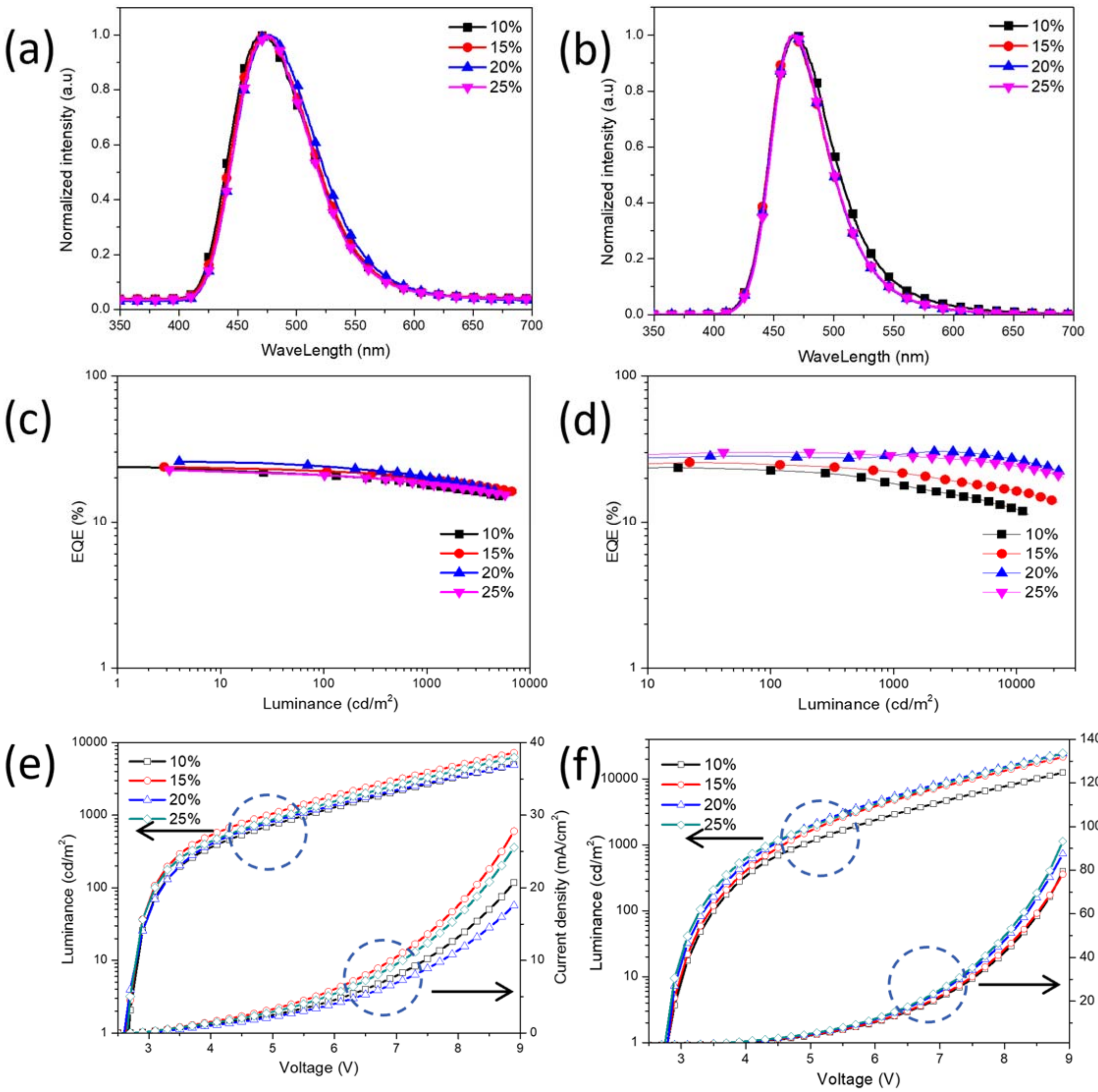

Figure S7. EL spectra of ***f*-ct9a** (a) and ***f*-ct9b** (b); EQE vs. luminance diagram of ***f*-ct9a** (c) and ***f*-ct9b** (d); J-V-L curves ***f*-ct9a** (e) and ***f*-ct9b** (f) in **Device I** with architecture: ITO/ HATCN (5nm)/ BCFN (30nm)/ SiCzCz (10nm)/ 65 wt% SiCzCz: 35 wt% SiTrzCz2: x wt% ***f*-ct9a** or ***f*-ct9b** (30nm) / mSiTrz (5nm) / DPPyA (30nm)/ LiF (0.5nm)/ Al (150nm).

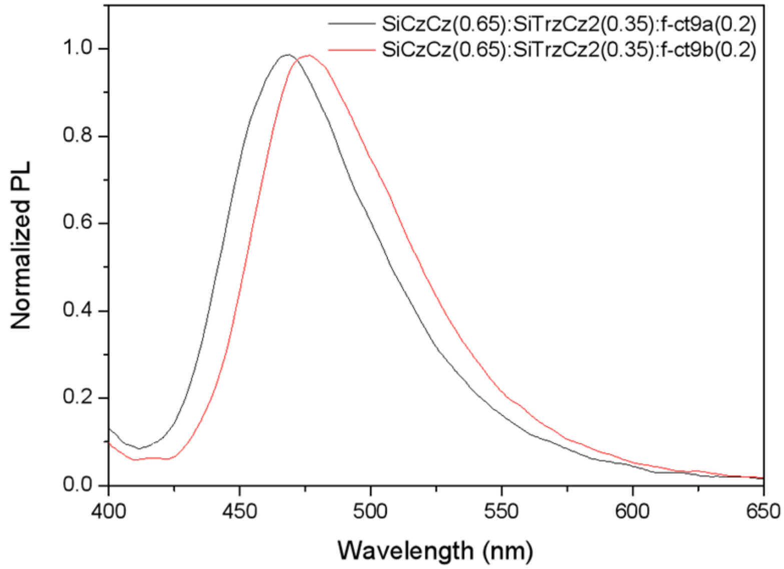

Figure S8. Emission spectra of ***f*-ct9a** and **b** with specified co-host and doping concentration at RT.

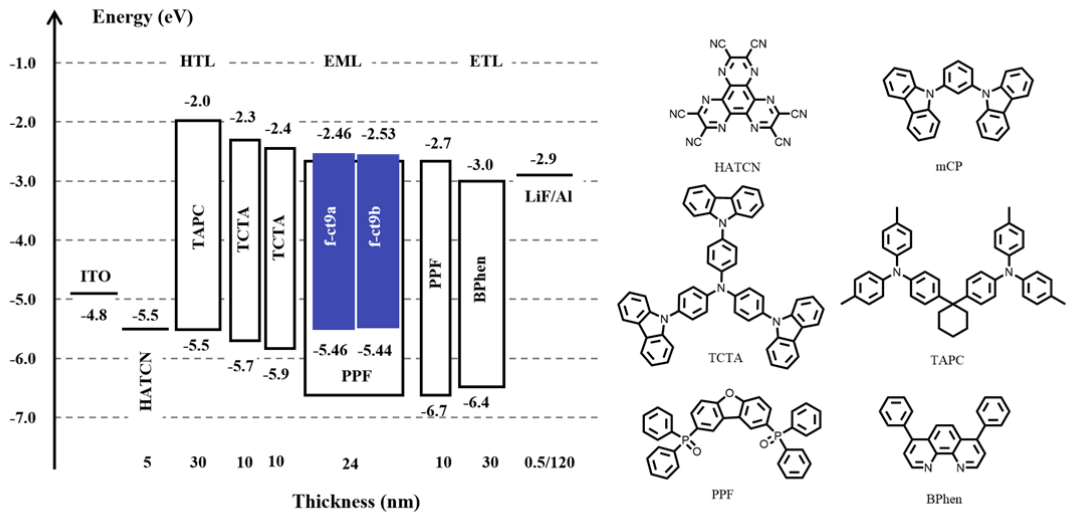

Figure S9. The structure of **Device II**, energy diagram and chemical structures of the materials used in the devices.

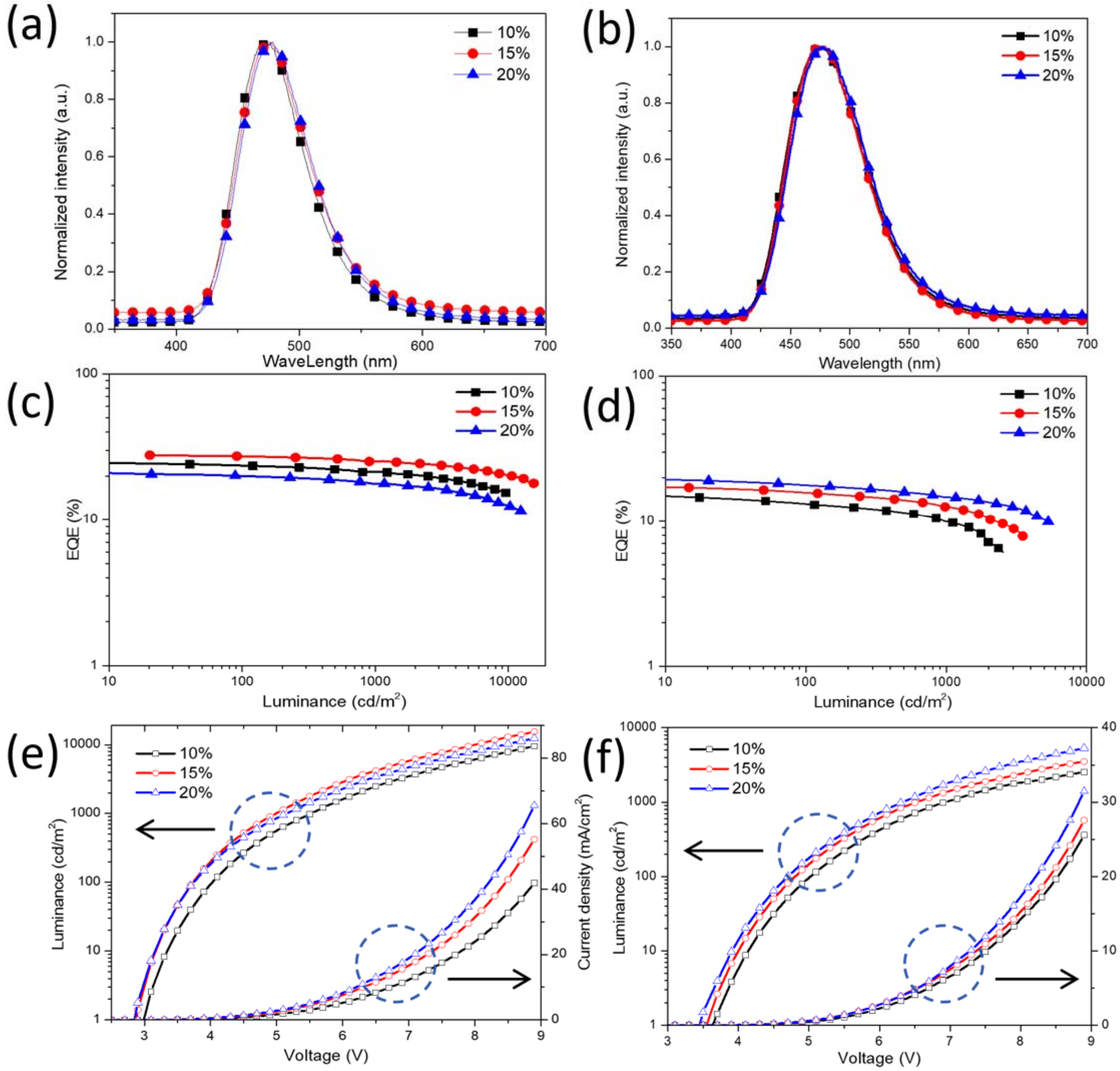

Figure S10. EL spectra of ***f*-ct9a** (a) and ***f*-ct9b** (b); EQE vs. luminance of ***f*-ct9a** (c) and ***f*-ct9b** (d); J-V-L curves ***f*-ct9a** (e) and ***f*-ct9b** (f) in **Device II** with architecture: ITO/ HATCN (5nm)/ TAPC (30nm)/ TCTA (10nm)/ PPF: x wt% ***f*-ct9a** or ***f*-ct9b** (24nm)/ PPF (10nm)/ BPhen (30nm)/ LiF (0.5nm)/ Al (120nm).

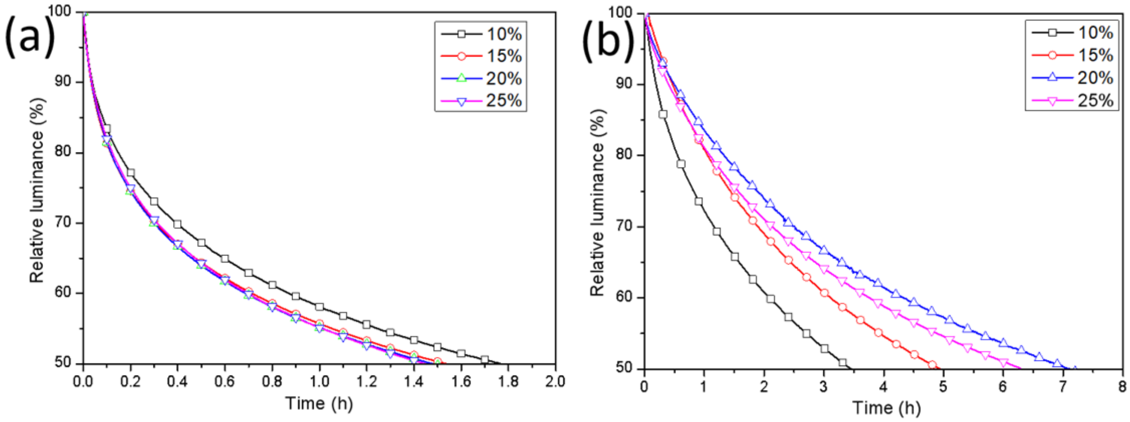

Figure S11. Relative luminance as a function of operational time of OLED Device I using **(a) *f*-ct9a** and **(b) *f*-ct9b** as emitter with L_0 = 1000 cd·m^{-2}.

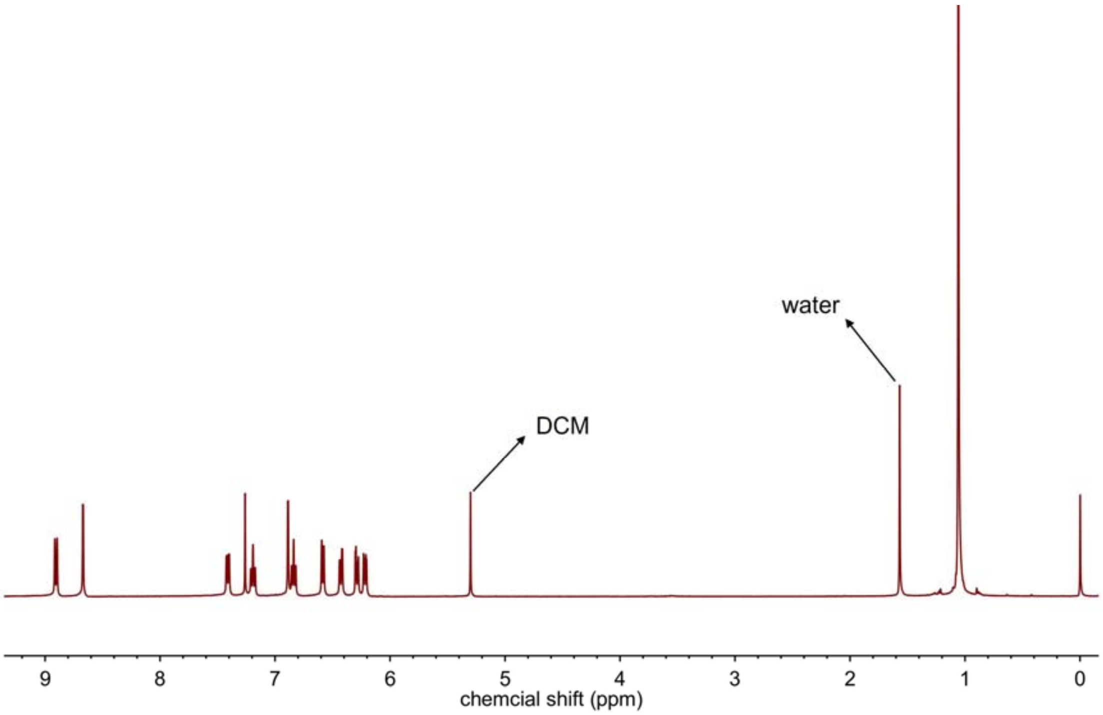

Figure S12. ^{1}H-NMR spectrum (400 MHz) of ***f*-ct9a** in $CDCl_3$ at 298 K.

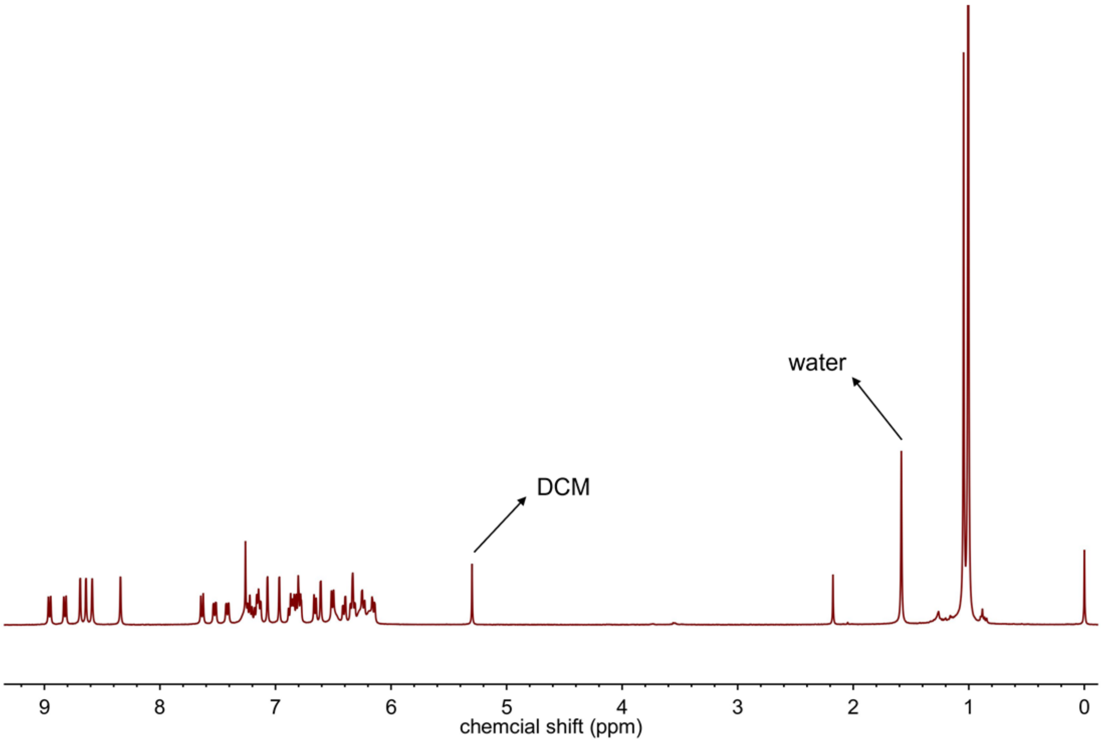

Figure S13. ^{1}H-NMR spectrum (400 MHz) of ***f*-ct9b** in $CDCl_3$ at 298 K.

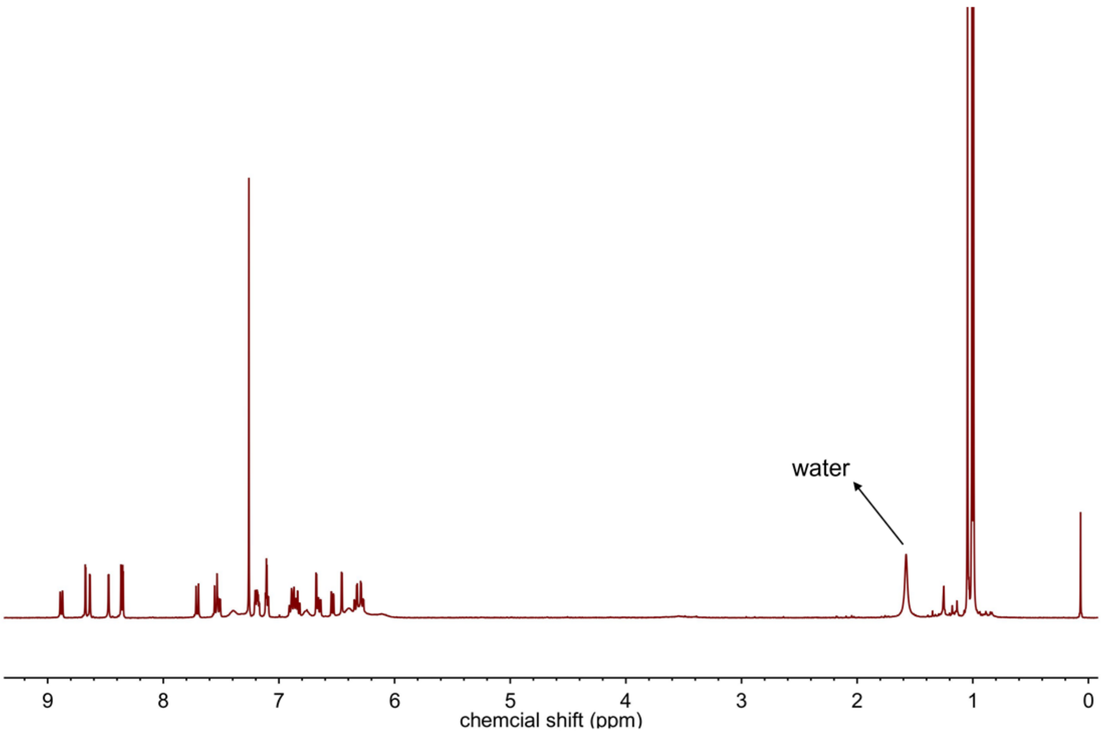

Figure S14. ^{1}H-NMR spectrum (400 MHz) of ***f*-ct9c** in $CDCl_3$ at 298 K.

Table S1. Photophysical data of Ir(III) carbene complexes ***f*-ct9a** and **b** in co-deposited SiCzCz:SiTrzCz2 thin films at RT.

	λ_{max} (nm) [a]	FWHM (nm) [b]	PLQY (%)[a]	τ_{obs} (µs) [a]	τ_{rad} (µs)	k_r (10^6 s^{-1})	k_{nr} (10^5 s^{-1})
SiCzCz(0.65):SiTrzCz2(0.35):***f*-ct9a**(0.2)	468	63	76	0.65	0.92	1.09	4.46
SiCzCz(0.65):SiTrzCz2(0.35):***f*-ct9b**(0.2)	476	65	94	1.04	1.35	0.74	2.21

[a] PL spectra, quantum yield and lifetime were recorded in doped co-host thin film at RT (20 wt.%) using integration sphere. [b] full width at half maximum.

Table S2. Electroluminescence Data of devices (**Device II**)

**Devices**		**OV [V]**	**EL [nm]**	**FWHM [nm]**	**CE [cd·A^{-1}]**	**PE [lm·W^{-1}]**	**EQE [%] (max / 1000 / 5000 cd·m^{-2})**	**CIE [x,y] @ 1000 cd·m^{-2}**
***f*-ct9a** conc.[%]	10 %	3.0	472	66	38.2	37.4	24.7/21.5/17.9	0.147, 0.208
	15 %	2.9	477	68	45.8	43.4	28.8/25.3/22.3	0.148, 0.227
	20 %	2.9	478	68	37.7	40.8	22.4/17.8/15.1	0.149, 0.239
***f*-ct9b** conc.[%]	10 %	3.7	475	78	25.5	21.7	15.8/9.90/-	0.149, 0.226
	15 %	3.6	479	75	30.4	25.8	17.1/12.5/-	0.147, 0.226
	20 %	3.5	476	77	36.7	32.9	20.9/14.6/10.0	0.149, 0.245

Table S3. Summary of reported performance of selected deep blue PHOLEDs based on similar Ir(III) carbene complexes

Dopants	λ_{max} [nm]	EQE [%]	Luminance [cd·m^{-2}]	CIE [x,y]	Ref.
***f*-ct9a**	478	25.9 / 20.0 / 16.1	max / 1000 / 5000	0.16, 0.26	This work
***f*-ct9b**	468	30.3 / 29.0 / 29.0	max / 1000 / 5000	0.14, 0.17	This work
***mer*-Ir(pmp)$_3$**	465	14.4 / 13.3 / -	max / 1000 / -	0.16, 0.15	[12]
***f*-tpb1**	472	13.5 / 12.0[a] / 11.0[a]	max / 1000 / 5000	0.15, 0.23	[13]
**Ir(cb)$_3$**	468	17.1 / 13.5 / -	max / 200 / --	0.14, 0.19	[14]

[a] EQE estimated values based on the graphic illustraction (not provided by author).

References

[1] a) C. Lee, W. Yang, R. G. Parr, *Phys. Rev. B* **1988**, *37*, 785-789; b) A. D. Becke, *J. Chem. Phys.* **1993**, *98*, 5648-5652; c) F. Weigend, R. Ahlrichs, *Phys. Chem. Chem. Phys.* **2005**, *7*, 3297-3305; d) F. Weigend, *Phys. Chem. Chem. Phys.* **2006**, *8*, 1057-1065; eS. Grimme, S. Ehrlich, L. Goerigk, *J. Comput. Chem.* **2011**, *32*, 1456-1465.

[2] M. J. Frisch, G. W. Trucks, H. B. Schlegel, G. E. Scuseria, M. A. Robb, J. R. Cheeseman, G. Scalmani, V. Barone, B. Mennucci, G. A. Petersson, H. Nakatsuji, M. Caricato, X. Li, H. P. Hratchian, A. F. Izmaylov, J. Bloino, G. Zheng, J. L. Sonnenberg, M. Hada, M. Ehara, K. Toyota, R. Fukuda, J. Hasegawa, M. Ishida, T. Nakajima, Y. Honda, O. Kitao, H. Nakai, T. Vreven, J. A. Montgomery, J. E. Peralta, F. Ogliaro, M. Bearpark, J. J. Heyd, E. Brothers, K. N. Kudin, V. N. Staroverov, R. Kobayashi, J. Normand, K. Raghavachari, A. Rendell, J. C. Burant, S. S. Iyengar, J. Tomasi, M. Cossi, N. Rega, J. M. Millam, M. Klene, J. E. Knox, J. B. Cross, V. Bakken, C. Adamo, J. Jaramillo, R. Gomperts, R. E. Stratmann, O. Yazyev, A. J. Austin, R. Cammi, C. Pomelli, J. W. Ochterski, R. L. Martin, K. Morokuma, V. G. Zakrzewski, G. A. Voth, P. Salvador, J. J. Dannenberg, S. Dapprich, A. D. Daniels, Ö. Farkas, J. B. Foresman, J. V. Ortiz, J. Cioslowski, D. J. Fox, *Gaussian 16, Revision C.01; Gaussian Inc.* **2016**, Wallingford, CT.

[3] a) S. Miertuš, E. Scrocco, J. Tomasi, *Chem. Phys.* **1981**, *55*, 117-129; b) S. Miertuš, J. Tomasi, *Chem. Phys.* **1982**, *65*, 239-245.

[4] a) C. Adamo, D. Jacquemin, *Chem. Soc. Rev.* **2013**, *42*, 845-856; b) A. D. Laurent, C. Adamo, D. Jacquemin, *Phys. Chem. Chem. Phys.* **2014**, *16*, 14334-14356.

[5] R. L. Martin, *J. Chem. Phys.* **2003**, *118*, 4775-4777.

[6] T. Lu, F. Chen, *J. Comput. Chem.* **2012**, *33*, 580-592.

[7] F. L. Hirshfeld, *Theo. Chim. Acta* **1977**, *44*, 129-138.

[8] B. de Souza, G. Farias, F. Neese, R. Izsák, *J. Chem. Theory Comput.* **2019**, *15*, 1896-1904.

[9] a) E. van Lenthe, E. J. Baerends, J. G. Snijders, *J. Chem. Phys.* **1993**, *99*, 4597-4610; b) E. van Lenthe, E. J. Baerends, J. G. Snijders, *J. Chem. Phys.* **1994**, *101*, 9783-9792.

[10] F. Neese, F. Wennmohs, U. Becker, C. Riplinger, *J. Chem. Phys.* **2020**, *152*, 224108.

[11] C. C. Pye, T. Ziegler, *Theor. Chem. Acc.* **1999**, *101*, 396-408.

[12] J. Lee, H. F. Chen, T. Batagoda, C. Coburn, P. I. Djurovich, M. E. Thompson, S. R. Forrest, *Nat Mater* **2016**, *15*, 92-98.

[13] X. Yang, X. Zhou, Y. X. Zhang, D. Li, C. Li, C. You, T. C. Chou, S. J. Su, P. T. Chou, Y. Chi, *Adv Sci (Weinh)* **2022**, *9*, e2201150.

[14] A. Maheshwaran, V. G. Sree, H.-Y. Park, H. Kim, S. H. Han, J. Y. Lee, S.-H. Jin, *Advanced Functional Materials* **2018**, *28*.
